# Dynamic participation in local energy communities with peer-to-peer trading

**DOI:** 10.12688/openreseurope.14332.1

**Published:** 2022-01-11

**Authors:** Theresia Perger, Hans Auer

**Affiliations:** 1 Institute of Energy Systems and Electrical Drives, Energy Economics Group (EEG), TU Wien, Vienna, 1040, Austria

**Keywords:** Peer-to-peer trading, Energy communities, Willingness-to-pay, Bi-level programming, Open-source, Energy system modeling

## Abstract

**Background:** Energy communities and local electricity markets (e.g., as peer-to-peer trading) are on the rise due to increasingly decentralized electricity generation and favorable adjustment of the legal framework in many European countries.

**Methods: **This work applies a bi-level optimization model for dynamic participation in peer-to-peer electricity trading to determine the optimal parameters of new participants who want to join an energy community, based on the preferences of the members of the original community (e.g., environmental, economic, or mixed preference). The upper-level problem chooses optimal parameters by minimizing an objective function that includes the prosumers' cost-saving and emission-saving preferences, while the lower level problem maximizes community welfare by optimally allocating locally generated photovoltaic (PV) electricity between members according to their willingness-to-pay. The bi-level problem is solved by transforming the lower level problem by its corresponding Karush-Kuhn-Tucker (KKT) conditions.

**Results: **The results demonstrate that environment-oriented prosumers opt for a new prosumer with high PV capacities installed and low electricity demand, whereas profit-oriented prosumers prefer a new member with high demand but no PV system capacity, presenting a new source of income. Sensitivity analyses indicate that new prosumers' willingness-to-pay has an important influence when the community must decide between two new members.

**Conclusions:** The added value of this work is that the proposed method can be seen as a basis for a selection process between a large number of potential new community members. Most important future work will include optimization of energy communities over the horizon several years.

## Nomenclature

**Table:** 

*Sets*	
*t* ∈ = {1, . . . , *T*}	Time steps
*i* ∈ ℐ = {1, . . . , *N* + *n*}	Index of all prosumers
*i* ∈ ℐ _*old*_ = {1, . . . , *N*}	Index of old prosumers
*i* ∈ ℐ _*new*_ = { *N* + 1, . . . , *N* + *n*}	Index of new prosumers
*Parameters*	
$q_{i,t}^{load}$	Demand of prosumer *i* (kWh)
$q_{i,t}^{PV}$	PV generation of prosumer *i* (kWh)
$load_{i}^{max}$	Maximum annual demand of prosumer *i* ∈ ℐ _*new*_ (kWh)
$load_{i}^{min}$	Minimum annual demand of prosumer *i* ∈ ℐ _*new*_ (kWh)
$PV_{i}^{max}$	Maximum peak PV generation of prosumer *i* ∈ ℐ _*new*_ (kW)
$PV_{i}^{min}$	Minimum peak PV generation of prosumer *i* ∈ ℐ _*new*_ (kW)
$SoC_{i}^{max}$	Capacity of prosumer *i*’s battery (kWh)
$q_{i}^{B_{max}}$	Maximum (dis)charging power of prosumer *i*’s battery (kW)
*η ^B^*	Efficiency of the batteries
*w _j_*	Prosumer *j*’s weighting factor for marginal emissions (EUR/tCO _2_)
*d _ij_*	Distance factor between prosumer *i* and *j* (∈ [0, 1])
*wtp _i,j,t_*	Willingness-to-pay of prosumer *j* (EUR/kWh)
*α _i_*	Upper-level preference factor of prosumer *i* within range (∈ [0, 1])
$p_{t}^{G_{in}}$	Average spot market electricity price (EUR/kWh)
$p_{t}^{G_{out}}$	Retailer’s electricity price (EUR/kWh)
*e _t_*	Marginal emissions from the grid (tCO _2_/kWh)
*Decision variables*	
*load _i_*	Annual demand of prosumer *i* ∈ ℐ _*new*_ (kWh)
*PV _i_*	Installed PV capacity of prosumer *i* ∈ ℐ _*new*_ (kW)
*b _i_*	Binary decision variable of prosumer *i* ∈ ℐ _*new*_
$q_{i,t}^{G_{in}}$	Purchase of prosumer *i* from the grid (kWh)
$q_{i,t}^{G_{out}}$	Sales from prosumer *i* to the grid (kWh)
$q_{i,j,t}^{share}$	Purchase of prosumer *j* from prosumer *i* (kWh)
$q_{i,t}^{B_{in}}$	Charging of prosumer *i*’s battery (kWh)
$q_{i,t}^{B_{out}}$	Discharging of prosumer *i*’s battery (kWh)
*SoC _i,t_*	State of charge of prosumer *i*’s battery (kWh)

## 1 Introduction

### 1.1 Motivation

The increasing number of photovoltaic (PV) systems in our energy system leads to a high share of decentralized production. Households or small businesses that were previously considered consumers only now have the opportunity to become prosumers. To go beyond individual self-consumption of single prosumers, collective forms of self-consumption take advantage of load aggregation to further optimize the use of resources (Frieden *et al.* ^1^). By sharing or trading self-generated electricity within a certain framework, for example in energy communities, prosumers become active participants in the energy system. There are also opportunities to form local, decentralized electricity markets. In peer-to-peer trading, participants trade electricity directly with other participants, the "peers" (Bjarghov *et al.* ^2^, Sousa *et al.* ^3^, and Tushar *et al.* ^4^). Peer-to-peer trading allows participants to increase their consumption of locally generated clean energy and to increase flexibility. Prosumers usually seek to maximize their economic or environmental benefits; hence, a fair pricing mechanism and trust in the community are crucial in this aspect. Furthermore, peer-to-peer trading and energy communities are opportunities to create new sustainable business models (F.G. Reis *et al.* ^5^). When transitioning toward a world with a high share of renewables, it can be assumed that local electricity markets, such as peer-to-peer trading or pool markets, are more established and sufficient regulatory framework exists.

### 1.2 Core objective and research question

The core objective of this research is to investigate and optimize energy communities, wherein prosumers trade self-generated PV electricity with one another (peer-to-peer trading), including members’ entry and exit over time. The research question is the following: How would an existing energy community collectively choose an optimal new member/prosumer to engage in peer-to-peer trading? With the model developed in this work, it is possible to (i) choose between different prosumers, and (ii) choose the desired parameters of a new prosumer.

### 1.3 Method applied

The method applied is based on a linear optimization model for local energy communities that was previously developed by the authors in Perger *et al.* ^6^. The objective of this model is to optimally allocate electricity trades between community members considering each prosumer’s individual willingness-to-pay for locally generated PV electricity. To answer the research question, the model is extended to a bi-level optimization problem.

The model developed is an operating model rather than an investment model, assuming that in the future (i) many people will already have PV modules and (ii) PV systems will be "mainstream products" and therefore installing a PV system is a low barrier for those interested in joining a local energy market. In particular, a community’s new member selection and decision-making process is the subject of interest.

### 1.4 Structure of the paper

The next Section 2 presents a comprehensive literature review of local energy markets, peer-to-peer trading mechanisms, and the regulatory framework. Section 2 concludes with the paper’s contributions beyond state-of-the-art. Section 3 explains the methodology and modeling approach, and presents the data and assumptions of the case study. Section 4 presents the results of the case study, followed by a sensitivity analysis in Section 5. A conclusion and the outlook for future research needs in Section 6 complete the paper.

## 2 State-of-the-art and progress beyond

This Section provides a review and discussion of recent, relevant scientific literature regarding energy communities and peer-to-peer trading. Section 2.1 reviews state-of-the-art peer-to-peer trading modeling approaches. Section 2.2 gives an overview of related research on policy and legislation, with focus on Europe, and on the social aspects of energy communities. Section 2.3 presents this paper’s contribution beyond state-of-the-art.

### 2.1 Peer-to-peer trading models in literature

A comprehensive review of existing literature and modeling approaches in the field of peer-to-peer trading is presented in Soto *et al.* ^7^. Most peer-to-peer trading models consider consumers, prosumers, an energy sharing coordinator, and an electricity supplier/retailer. There are different approaches to implementing the energy exchange and negotiation processes. In Soto *et al.* ^7^, they are categorized into trading platforms, blockchain, game theory, simulation, optimization, and algorithms. Different non-cooperative game theory approaches for peer-to-peer trading of prosumers in microgrids with PV systems and battery storage are developed in Paudel *et al.* ^8^ and Zhang *et al.* ^9^. A canonical coalition game for peer-to-peer trading is presented in Tushar *et al.* ^10^, while Fleischhacker *et al.* ^11^ compares a Stackelberg game with a welfare maximization model for PV sharing in multi-apartment buildings. Continuous double auctioning models for peer-to-peer trading are developed in Li and Ma ^12^, Chen *et al.* ^13^, and Lin *et al.* ^14^.

To decrease aggregated peak load, Bjarghov *et al.* ^15^ developed a peer-to-peer trading capacity market formulated as a mixed complementarity problem (MCP). Sharing energy in a community-based market structure including fairness indicators is proposed in Moret and Pinson ^16^. Jiang *et al.* ^17^ presents a two-stage optimization approach, including social utility maximization in the first stage and payment bargaining in the second stage. Comparing three different models, Henriquez-Auba *et al.* ^18^ found that a sharing economy model in which PV generation is traded among firms in a local spot market is a plausible pathway to maintaining and accelerating investments in PV systems, considering that feed-in programs are likely to be phased-out in the near future. Peer-to-peer markets with product differentiation are introduced in Sorin *et al.* ^19^. In Hashemipour *et al.* ^20^, virtual local energy markets with dynamic allocation of clusters that change on a daily basis are developed. Electric vehicles are pooled into the market to further increase flexibility.

Potential congestion and voltage problems in the distribution network considering the increasing penetration of Distributed Energy Resources are addressed in recent papers on peer-to-peer trading. For example, Dynge *et al.* ^21^ analyze the impact of the low voltage grid on local markets. As the physical distribution network is used for trades in local electricity markets, a market clearing approach considering network fees and power losses is introduced in Paudel *et al.* ^22^. The Euclidean distance of the distribution network between peers is included as grid-related costs using a product differentiation method in Orlandini *et al.* ^23^. Another product differentiation approach is presented in Khorasany *et al.* ^24^ in which network constraints are considered using a power transfer distribution factor to represent the contribution of transactions in the line flows. Considering electrical distances between prosumers, Guerrero *et al.* ^25^ include a shortest path algorithm in their peer-to-peer market design and compare stable-matching and continuous double auction allocation mechanisms. An optimization problem solving matching between peers, including least-cost energy path algorithms, is proposed by Jogunola *et al.* ^26^.

A selection of real-life implementations of peer-to-peer trading examples includes Piclo (Piclo ^27^) in the UK, The Brooklyn Microgrid (Microgrid ^28^ and Mengelkamp *et al.* ^29^) in the US, Vandebron (Vandebron ^30^) in the Netherlands, and the sonnenCommunity (sonnenGroup ^31^) in Austria, Germany, Italy, and Switzerland. A detailed review of existing peer-to-peer trading projects including those mentioned is found in Zhang *et al.* ^32^.

### 2.2 Participation in local energy markets or communities from a policy and social perspective

A number of legal instruments are included in the European Union’s Clean Energy Package Directorate-General for Energy (European Commission) ^33^ introducing the legal framework to establish the sharing/trading of self-generated electricity and to initiate economic incentives for its practice. EU member states are obliged to enable the entrance of these active participants into markets. Furthermore, the Clean Energy Package introduced a definition of *peer-to-peer trading*. Nevertheless, many regulatory aspects of peer-to-peer trading remain unclear. A review of current European policies, legislation, and possible legal issues related to peer-to-peer trading and energy communities in electricity markets is presented in de Almeida *et al.* ^34^. The European guidelines of the Clean Energy Package as transposed into Austrian law is analyzed in Fina and Fechner ^35^.

Azarova *et al.* ^36^ analyze how to design a Renewable Energy Community (REC) to increase social acceptance, finding that acceptance for solar farms and power-to-gas infrastructure is high, mixed for wind farms, and low for gas power plants and power lines. To gain more knowledge regarding individuals’ willingness to participate in energy communities, using regression analysis, Koirala *et al.* ^37^ conducted a survey in the Netherlands to determine the importance of factors such as environmental concerns, renewable acceptance, community trust, and resistance (among others). According to the survey, perceived barriers for participation include lack of time, financial reasons, satisfaction with the status quo of the energy system, and no trust in the neighborhood.

According to the analysis in Hackbarth and Löbbe ^38^ focusing on intentions of private households to participate in peer-to-peer trading mechanisms in Germany, highly interested potential participants exhibit environmental rather than economic preferences, and are drawn to innovative pricing schemes. Soeiro and Ferreira Dias ^39^ found that trust is a key component and that citizens recognize the added non-monetary values of renewable energy communities.

In contrast to Germany and the Netherlands, there is a delay in the development and integration of RECs in Southern European countries. Using a survey in Spain and Portugal, Soeiro and Dias ^40^ aims to understand the motivations of members in energy communities.

The inclusion of vulnerable consumers in the energy transition, who are generally underrepresented in REC projects, is discussed in Hanke and Lowitzsch ^41^. The enabling framework to support inclusion remains rather unclear and should not languish as an idea on paper; therefore, lawmakers and policymakers should develop incentives targeting both RECs and individual vulnerable consumers.

Regarding peer-to-peer trading concepts in particular, Reis *et al.* ^42^ developed a multi-agent framework to model peer-to-peer electricity within energy communities with an emphasis on vulnerable consumers and members’ economic outcomes, considering fairness in the distribution of energy resources. Fair revenue sharing and exit clauses are examined in Fioriti *et al.* ^43^, to identify the optimal sizing of energy communities.

### 2.3 Contribution beyond state-of-the-art

To date, there has been minimal analysis of dynamic participation (entry and exit) in energy communities over time. This is where the present paper picks up. The contribution of the following analyses beyond state-of-the-art is summarized as follows:

- A method is developed based on the peer-to-peer allocation mechanism presented in 6 to optimize energy communities with peer-to-peer trading over the years, including members’ entry and exit.
- A novel peer-to-peer model is proposed that simultaneously provides (i) an allocation mechanism for electricity trades between members and (ii) a new member’s selection process. Both (i) and (ii) take the prosumers’ individual preferences into account.
- The selection process, which is of particular interest, operates from the perspective of the community members, wherein community members are searching for "optimal fitting participants" as opposed to optimal technologies.
- The insights gained from the results and sensitivity analyses expand the understanding of the importance of participants’ individual preferences. These insights offer practical considerations to help establish stable and prosperous local energy communities.

## 3 Methods

In this Section, the methodology and modeling approach are described. The framework of the study explained in Section 3.1 is followed by a detailed description of the optimization problem in Section 3.2. Data and assumptions are presented in Section 3.3, and Section 3.4 introduces the verification of the proposed modeling approach. 

### 3.1 Dynamic participation in energy communities

**Figure 1.**
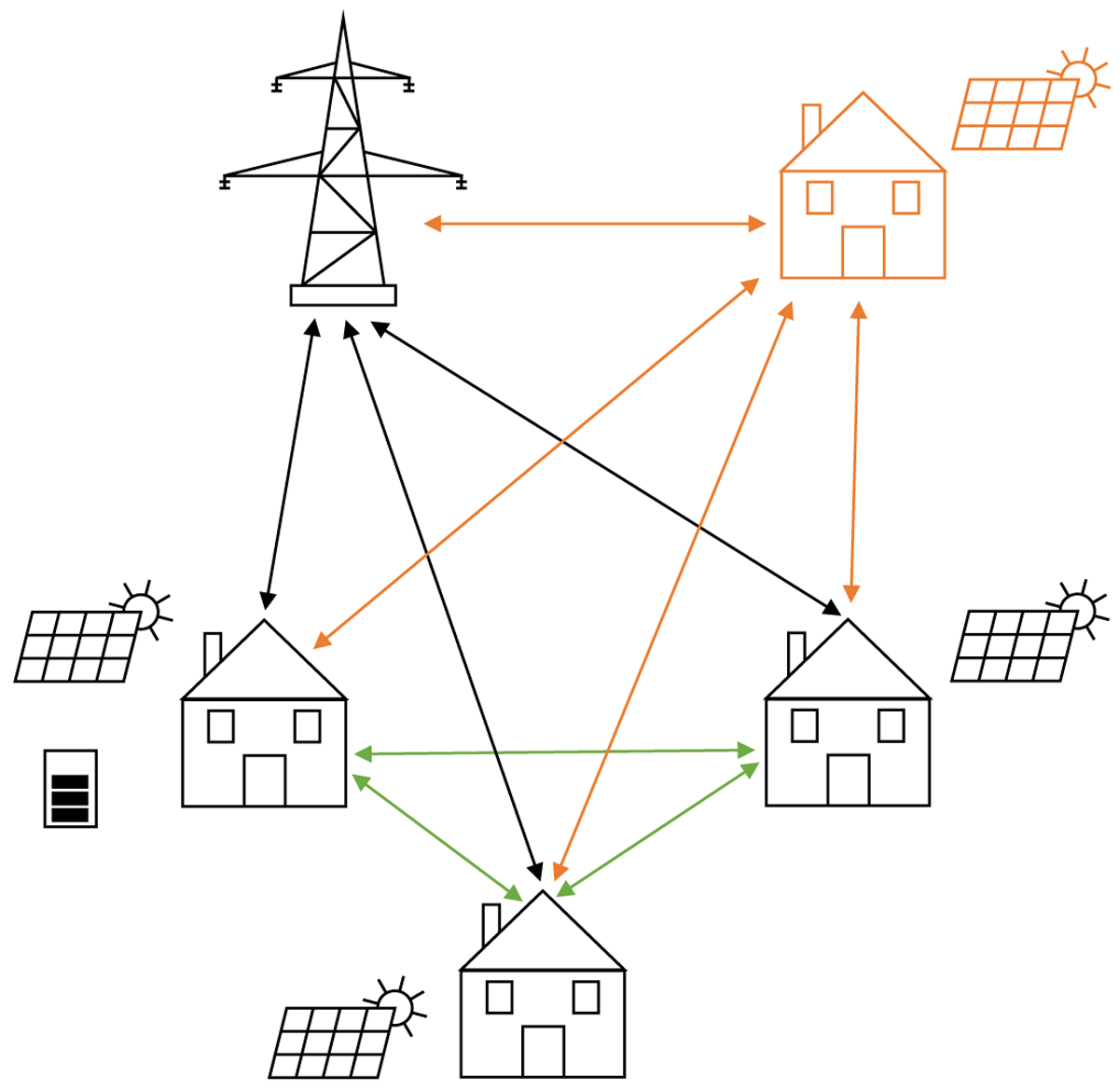
Sketch of the framework of the modeling approach.

***3.1.1 Modeling framework.*** The framework of the modeling approach is a peer-to-peer electricity trading concept in a local energy community. Prosumers (or consumers or producers) join on a voluntary basis and exchange PV electricity generated by community members with one another. Figure 1 presents the basic idea of the peer-to-peer trading concept in this paper. All members are connected to the public distribution grid to be able to cover the community’s residual load, to feed in the surplus PV electricity, and to trade with the other community members (green arrows). Participants in the community are either households or small-to-medium-sized enterprises. The technology portfolio includes PV systems and battery energy storage systems (BESSs). In addition, each prosumer has an individual willingness-to-pay for PV electricity generated by community members, which determines the allocation of the peer-to-peer trading.

The aim of this work is to optimize the dynamic participation of prosumers in an energy community; hence, changes in the set-up of members over time (i.e., exit/entry). In Figure 1, the orange parts represent a new member joining the community.

In this context, new prosumers are characterized by (i) electricity load/demand, (ii) electricity generation (PV system and BESS size), and (iii) consumer-type (household or small business). Other characteristics include electrical distance from the other community members, the minimum and maximum number of new prosumers, and the length of binding contracts with the community. The latter is out of scope for this paper.

**Figure 2.**
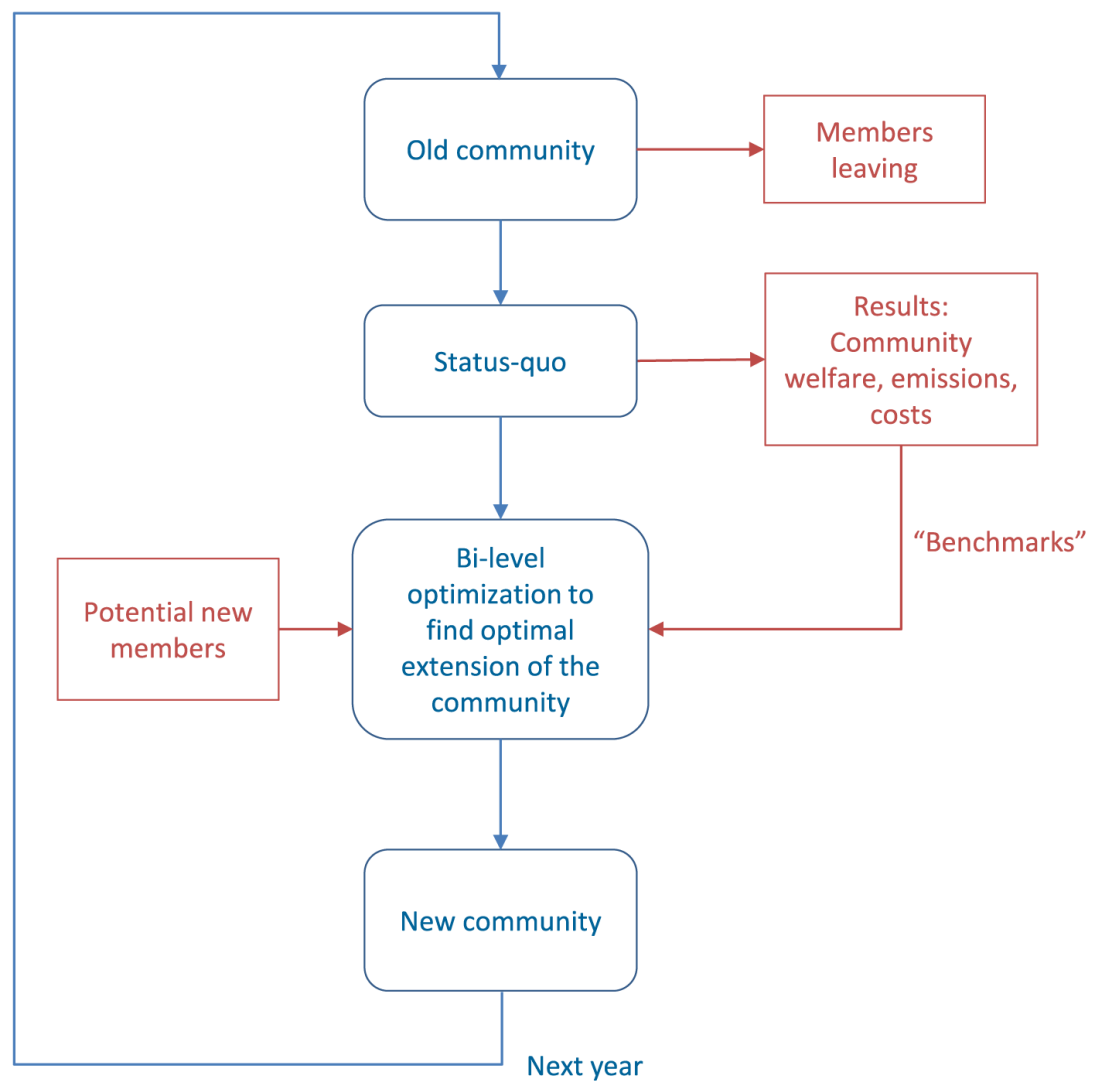
Flow chart of the proposed methodology.

***3.1.2 Flow chart.*** The minimum length of a contract for prosumer participation in energy communities is assumed to be one year. There is a deadline each year; until then, members can decide to leave the community in the next contract period, or decide to stay and extend the contract for another year. In the meantime, prospective new members can declare interest in joining the community until the annual deadline. The flow chart in Figure 2 shows the process that is suggested to optimize dynamic participation in energy communities over a horizon of several years.

- The starting point is the "old" community, where some members leave at the end of their contract period.
- The status quo of the remaining members is then captured. Previous analyses of peer-to-peer electricity trading under the consideration of prosumers’ willingness-to-pay demonstrate two important characteristics for a community and its members: Overall community welfare ^1^, and the annual emissions and costs of each member. These indicators are obtained by solving a linear program (see the model presented in Perger *et al.* ^6^) to maximize community welfare of the original community configuration. The annual costs and emissions are then used as "benchmarks" for the optimization process.
- After decisions about leaving, staying, or joining the community are made by all existing and potential new members, a bi-level optimization problem is solved to determine the optimal configuration of new prosumers. The lower level problem is linear community welfare maximization that was applied to the original community in the previous step to obtain benchmarks. The upper-level problem determines which potential members are selected by the community, and subsequently, the new prosumers’ parameters (annual electricity demand and peak capacity of the installed PV systems). ^2^
- Finally, the new community is defined and the process repeats in the next year.

In this work, the implementation of the proposed method is shown for one period (year) in order to focus on the selection process of the community that is conducted using the bi-level optimization approach.

### 3.2 Mathematical formulation of the optimization problem

***3.2.1 Willingness-to-pay of prosumers.*** Prosumers’ individual willingness-to-pay determines how PV generated electricity is distributed among community members. The baseline of the willingness-to-pay is the retail electricity price, $p_{t}^{G_{in}}$ , and an individual CO _2_-price, *w _j_*, is added on top that relates to the prosumer’s preference for reducing emissions from electricity consumption. In addition, there is also a preference, *d _i,j_* ∈ [0, 1], to buy more locally (i.e., buying from a prosumer with the shortest electrical distance). The willingness-to-pay of prosumer *j* at time *t* to buy from prosumer *i*, *wtp _i,j,t_* , is as follows:


$$\;wtp_{i,j,t}=p_{t}^{G_{in}}+w_{j}(1-d_{i,j})\cdot e_{t}.\;(1)$$

The emissions from the grid, *e _t_*, are represented as a time series using the greenhouse gases emitted into the wider electricity system by the marginal power plant; hence, they are also known as marginal emissions. The local energy community is assumed to be a price taker in the wider electricity system.

***3.2.2 Community welfare.*** In this work, the aim of peer-to-peer electricity trade is to maximize community welfare, which is defined in two parts. Part I of community welfare measures the optimal resource allocation at the community level, maximizing the community’s self-consumption as a whole. Part II optimally assigns PV generated electricity to each member in consideration of their individual willingness-to-pay; thus, part II represents peer-to-peer trading from one prosumer to another, $q_{i,j,t}^{share}$ . Community welfare ( *CW*) is defined as following:


$$CW=\underset{\text{I}}{\underset{︸}{\sum_{t\in ,i\in ℐ}p_{t}^{G_{out}}q_{i,t}^{G_{out}}-\sum_{t\in ,i\in ℐ}p_{t}^{G_{in}}q_{i,t}^{G_{in}}}}+\underset{\text{II}}{\underset{︸}{\sum_{t\in ,i,j\in ℐ}wtp_{i,j,t}q_{i,j,t}^{share}.}}\;(2)$$

***3.2.3 Prosumers’ cost-emission function.*** To evaluate the impact of new prosumers on original prosumers, the following functions are defined:


$$\;\text{Δ}costs_{i}=costs_{i}-costs_{i,old},\;(3)$$

$$\;\text{Δ}emissions_{i}=emissions_{i}-emissions_{i,old}.\;(4)$$

Equation (3) is the deviation of prosumer *i*’s annual costs within the new community set-up compared to the previous status quo. Similar to Eq. (3), Eq. (4) represents prosumer *i*’s annual emission increase or decrease. The cost-emission function *CE* is defined next.


$$\;CE=\sum_{i\in {ℐ}_{old}}\alpha_{i}\text{Δ}costs_{i}+(1-\alpha_{i})\text{Δ}emissions_{i}\;(5)$$

Similar to Pareto-optimization, a weighting factor *α _i_* ∈ [0, 1] is introduced for each prosumer to choose individually. Therefore, *α _i_* determines whether more emphasis is placed on minimizing costs or emissions. By choosing an individual *α _i_*, prosumers can express either a cost-saving or an emission-saving preference. Due to the absolute values of costs and emissions in Eq. (3) and ( 4), each prosumer’s changes count equally. The cost-emission function *CE* is the objective to be minimized in the optimization problem.

The costs of each member *i* of the community over a certain period are calculated as following:


$$\;\begin{array}{l} costs_{i}\;=\sum_{t\in }p_{t}^{G_{in}}q_{i,t}^{G_{in}}-\sum_{t\in }p_{t}^{G_{out}}q_{i,t}^{G_{out}}\\ \;+\sum_{t\in ,j\in ℐ}wtp_{j,i,t}q_{j,i,t}^{share}-\sum_{t\in ,j\in ℐ}wtp_{i,j,t}q_{i,j,t}^{share}, \end{array}\;(6)$$

where is the respective time period. The emissions over a certain time are:


$$\;emissions_{i}=\sum_{t\in }e_{t}q_{i,t}^{G_{in}}\;(7)$$

Only purchases from the grid are considered in the emissions calculations, because the production of PV electricity does not generate marginal emissions.

***3.2.4 Bi-level optimization problem.*** This model solves two main problems: (i) selecting the optimal electricity demand and PV capacity of new prosumers to fulfill certain requirements set by original community members, and (ii) maximizing community welfare, given the new prosumers’ parameters selected in (i). Subsequently, this problem can be formulated as a bi-level problem, wherein the leader anticipates the follower’s reaction. In the upper-level problem, the *leader*, of the bi-level problem represents (i) and its lower level, the *follower*, (ii).

The leader minimizes the cost-emission function *CE* with the continuous decision variables *load _i_* and *PV _i_*, and the binary decision variables *b _i_*, for all *i* ∈ ℐ _*new*_ (see Eq. (8a)). The decision variables have lower and upper bounds to ensure a reasonable solution of the model (see Eqs. (8b) and ( 8c)). The set of variables


$$Q_{i,t}=\{q_{i,t}^{G_{in}},q_{i,t}^{G_{out}},q_{j,i,t}^{share},q_{i,t}^{B_{in}},q_{i,t}^{B_{out}},SoC_{i,t}\}$$

are the lower level primal decision variables. The dual variables of the lower level problem are $\left\{\lambda_{i,t}^{load},\lambda_{i,t}^{PV},\lambda_{i,t}^{SoC}\right\}$ for equality constraints, $\left\{\mu_{i,t}^{SoC^{max}},\mu_{i,t}^{B_{in}^{max}},\mu_{i,t}^{B_{out}^{max}}\right\}$ for inequalities, and $\left\{\beta_{i,t}^{G_{in}},\beta_{i,t}^{G_{out}},\beta_{i,j,t}^{share},\beta_{i,t}^{SoC},\beta_{i,t}^{B_{in}},\beta_{i,t}^{B_{out}}\right\}$ for non-negativities. The objective function of the follower in Eq. (8e) maximizes community welfare. The equality constraints ( 8f)–( 8i) ensure that prosumer *i*’s electricity demand and PV generation are covered at all times. The upper-level decision variables are included in Eq. (8h) and (8i) for new prosumers. The state of charge of prosumer *i*’s BESS is defined in Eqs. (8j) and ( 8k), and other battery constraints in ( 8l)–( 8n). Non-negativity conditions are included in ( 8o).


$$\underset{\left\{load_{i},PV_{i},b_{i},Q_{i,t}\right\}}{\min}\;\sum_{i\in {ℐ}_{old}}\alpha_{i}\text{Δ}costs_{i}+\;(1-\alpha_{i})\;\text{Δ}emissions_{i}\;(8a)$$

subject to:


$$b_{i}\cdot load_{i}^{min}\;\leq load_{i}\leq b_{i}\cdot load_{i}^{max}\;\forall i\;\in \;{ℐ}_{new}\;(8b)$$

$$b_{i}\cdot PV_{i}^{min}\;\leq PV_{i}\leq b_{i}\cdot PV_{i}^{max}\;\forall i\;\in \;{ℐ}_{new}\;(8c)$$

$$\sum_{i\in {ℐ}_{new}}b_{i}\;=\;n\;(8d)$$

$$\underset{Q_{i,t}}{\max}\sum_{t\in ,i\in ℐ}p_{t}^{G_{out}}q_{i,t}^{G_{out}}-\sum_{t\in ,i\in ℐ}p_{t}^{G_{in}}q_{i,t}^{G_{in}}+\sum_{t\in ,i,j\in ℐ}wtp_{i,j,t}q_{i,j,t}^{share}\;(8e)$$

subject to:


$$q_{i,t}^{G_{in}}+q_{i,t}^{B_{out}}+\sum_{j\in ℐ}q_{j,i,t}^{share}-q_{i,t}^{load}=0\;(\lambda_{i,t}^{load})\;\forall i\in {ℐ}_{old},t\;(8f)$$

$$q_{i,t}^{G_{out}}+q_{i,t}^{B_{in}}+\sum_{j\in ℐ}q_{i,j,t}^{share}-q_{i,t}^{PV}=0\;(\lambda_{i,t}^{PV})\;\forall i\in {ℐ}_{old},t\;(8g)$$

$$q_{i,t}^{G_{in}}+q_{i,t}^{B_{out}}+\sum_{j\in ℐ}q_{j,i,t}^{share}-load_{i}q_{i,t}^{load}=0\;(\lambda_{i,t}^{load})\;\forall i\in {ℐ}_{new},t\;(8h)$$

$$q_{i,t}^{G_{out}}+q_{i,t}^{B_{in}}+\sum_{j\in ℐ}q_{i,j,t}^{share}-PV_{i}q_{i,t}^{PV}=0\;(\lambda_{i,t}^{PV})\;\forall i\in {ℐ}_{new},t\;(8i)$$

$$SoC_{i,t-1}+q_{i,t}^{B_{in}}\cdot \eta^{B}-q_{i,t}^{B_{out}}/\eta^{B}-SoC_{i,t}=0\;(\lambda_{i,t}^{SoC})\;\forall i,t>t_{0}\;(8j)$$

$$SoC_{i,t=t_{end}}+q_{i,t_{0}}^{B_{in}}\cdot \eta^{B}-q_{i,t_{0}}^{B_{out}}/\eta^{B}-SoC_{i,t_{0}}=0\;(\lambda_{i,t_{0}}^{SoC})\;\forall i,t=t_{0}\;(8k)$$

$$SoC_{i,t}-SoC_{i}^{max}\leq 0\;(\mu_{i,t}^{SoC^{max}})\;\forall i,t\;(8l)$$

$$q_{i,t}^{B_{in}}-q_{i}^{B^{max}}\leq 0\;(\mu_{i,t}^{B_{in}^{max}})\;\forall i,t\;(8m)$$

$$q_{i,t}^{B_{out}}-q_{i}^{B^{max}}\leq 0\;(\mu_{i,t}^{B_{out}^{max}})\;\forall i,t\;(8n)$$

$$\begin{array}{l} -q_{i,t}^{G_{in}},-q_{i,t}^{G_{out}},-q_{i,j,t}^{share},\\ -q_{i,t}^{B_{in}},-q_{i,t}^{B_{out}},-SoC_{i,t}\leq 0\;(\beta_{i,t}^{G_{in}},\beta_{i,t}^{G_{out}},\beta_{i,j,t}^{share},\beta_{i,t}^{SoC},\beta_{i,t}^{B_{in}},\beta_{i,t}^{B_{out}})\;\forall i,t \end{array}\;(8o)$$

with *i*, *j* ∈ ℐ and *t* ∈ .

A very common approach to solving a bi-level optimization problem is the transformation to a mathematical program with equilibrium constraints (MPEC); see Ruiz *et al.* ^44^. The lower level problem ( Eqs. (8e)–( 8o)) is reformulated by its corresponding Karush-Kuhn-Tucker (KKT) conditions, and can be classified as a mixed complementarity problem (MCP) or equilibrium problem, which is parameterized by the leader’s decision variables (Dempe and Kue ^45^). The resulting optimization problem is single-level, and it is linear except for binary variables and complementarity constraints. The derivation of the KKT conditions is presented in detail in A. The resulting complementarity conditions are then transformed into a mixed integer linear program (MILP) using the Fortuny-Amat method (see A.3), also known as the "Big-M approach" (Fortuny-Amat and McCarl ^46^, Fischetti *et al.* ^47^, and Pineda *et al.* ^48^).

### 3.3 Data and assumptions

***3.3.1 Model implementation.*** The model is implemented using Python (version 3.7.2; see Van Rossum and Drake ^49^) using the Pyomo package (version 5.7.3; see Hart *et al.* ^50^ and 51), and Gurobi (version 9.0.0; see Gurobi Optimization, LLC ^52^) as a solver. Gurobi is a commercial solver. Alternatively, the problem can be solved with the open-source solver GNU Linear Programming Kit (GLPK). The model is available open source on GitHub ( *see Software availability*).

***3.3.2 Input data.*** To generate the results of a case study, a small community needs to be defined. The electricity demand of each member is obtained from the open-source tool LoadProfileGenerator (version 10.4.0; see Pflugradt and Muntwyler ^53^), which generates artificial data. Different household types categorized by living situation and demographics (single working person, elderly couple, family, etc.) are included in this study.

The PV generation data are obtained from a different open-source tool Renewables.ninja (version v1.3; see Pfenninger and Staffell ^54^, and Staffell and Pfenninger ^55^). PV systems’ irradiation data and electricity output are location-specific to Vienna, Austria.

While the existing community is characterized by specific input parameters, standardized profiles for the new prosumers are used as input data:

-
$q_{i,t}^{load}$ is a standardized load profile (H0 for household, G0 for standard business ^3^)), which is normalized to 1000kWh */*year. For example, a result of *load _i_* = 5 means that the optimal prosumer has an annual demand of 5000kWh */*year. The possible range is between 2000 – 8000kWh/year.
-
$q_{i,t}^{PV}$ is the generation profile of a 1kW _peak_ PV system facing South; hence, the decision variable *PV _i_* is a factor that upscales the PV system size. The possible range is between 0 – 5 kW _peak_.

**Figure 3.**
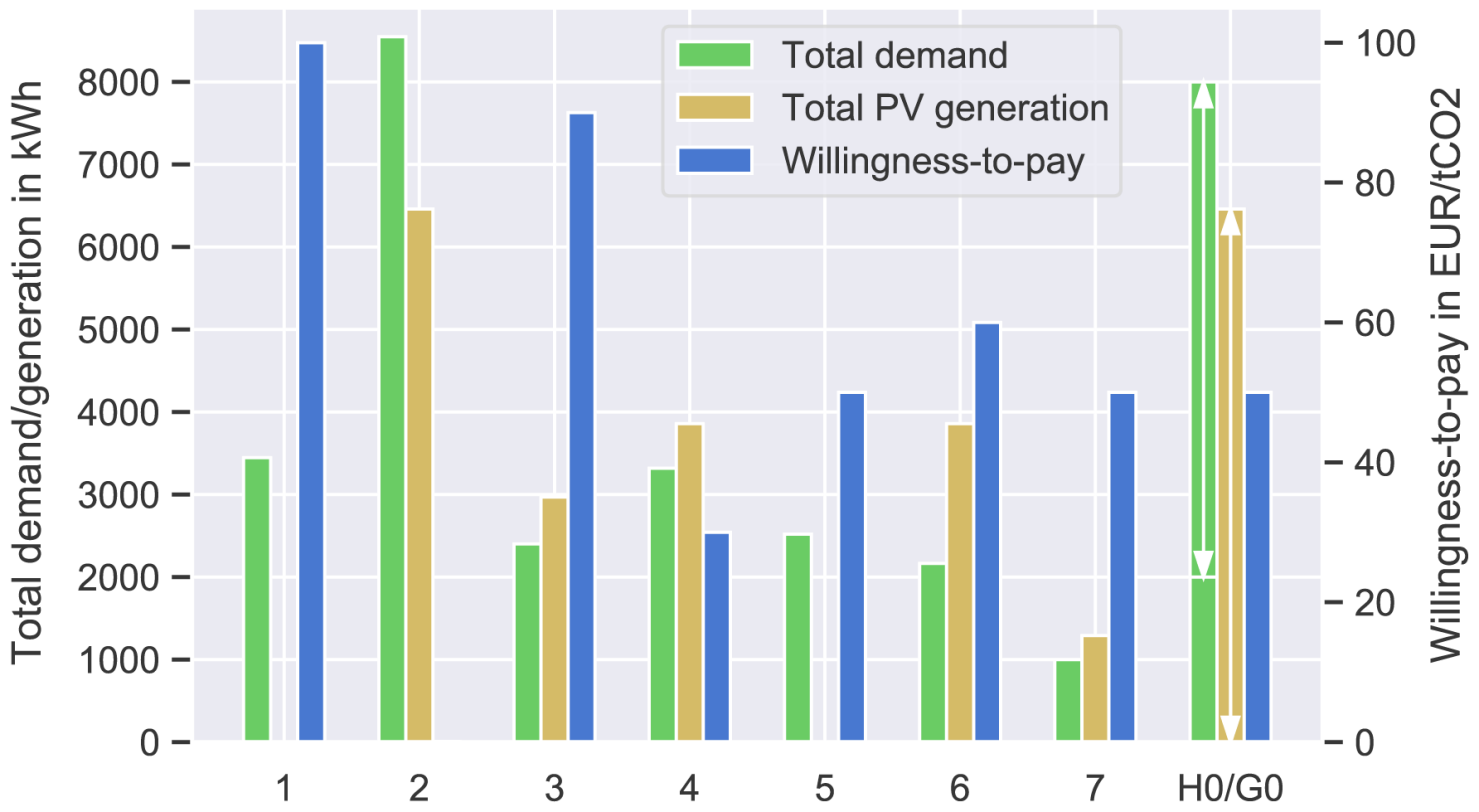
Annual electricity demand and photovoltaic (PV) generation of the prosumers (left axis); willingness-to-pay *w _j_* of each prosumer (right axis).

**Figure 4.**
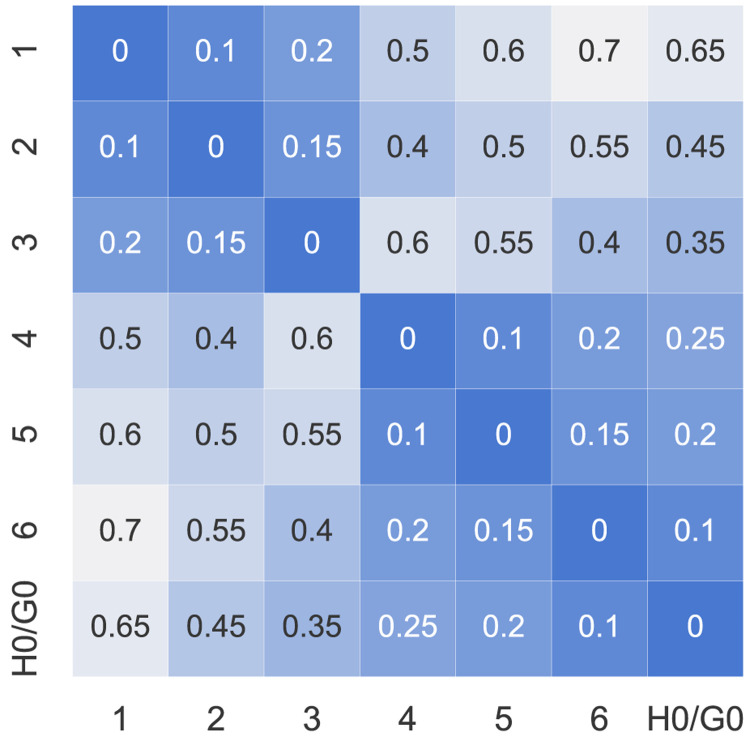
Distance factors *d _ij_* between the members (H0 and G0 represent the new prosumers).

A summary of the prosumers’ input data can be found in Figure 3 and in more detail in Table 1. The willingness-to-pay *w _i_* is arbitrarily assigned between the prosumers to cover a range between 0–100EUR */*tCO _2_. The electrical distance factors *d _ij_* ∈ [0, 1] can be represented by a symmetric matrix with diagonal elements all set to 0 (see Figure 4). The values assumed here are dummy values to represent electrical distances within a distribution network because the case study is artificial. The higher the value of *d _ij_*, the further the electrical distance between prosumer *i* and *j*.

**Table 1.** Parameters of the prosumers of the community ("-" indicates that a technology type is not included). The willingness-to-pay *w _i_* of the new prosumers (H0 and G0) is not optimized, but varied in a sensitivity analysis.

	Annual demand (kWh)	PV orientation	PV peak output (kW)	Storage capacity (kWh)	CO2 -price *w _i_* (EUR/tCO2)
Prosumer 1	3448	-	-	-	100
Prosumer 2	8548	South	5	-	0
Prosumer 3	2403	West	3	-	90
Prosumer 4	3320	South	3	3	30
Prosumer 5	2521	-	-	-	50
Prosumer 6	2167	South	3	-	60
Prosumer H0	2000 – 8000	South	0 – 5	-	0/50/100
Prosumer G0	2000 – 8000	South	0 – 5	-	0/50/100

Input data from the grid includes the following values: $p_{t}^{G_{in}}$ = 0.2EUR */*kWh (the average value of the 2019 Austrian retail electricity price; see 56) and $p_{t}^{G_{out}}$ = 0.04EUR */*kWh (average Austrian spot market price of 2019; see 57). Marginal emissions *e _t_* are hourly values obtained from 58 (Austrian-German spot market), and average hourly values are found in Figure 22 in the Appendix.

***3.3.3 Clustering in the time domain.*** Because MPECs are computationally expensive, an alternative approach is used to represent peer-to-peer trading within a community over a whole year. The input data that is available in hourly resolution for a whole year is transformed to three representative days using a k-means algorithm (Teichgraeber and Brandt ^59^) of the Python *tslearn* package (Tavenard *et al.* ^60^). The optimization model then determines the optimum using the three representative days considering the weight (each day represents a number of days of the year, which is then used to weight each representative day in the process of upscaling back to annual values; all three days represent the whole year) of each day in both the upper and lower level objective functions.

### 3.4 Validation of the bi-level modeling approach

In the bi-level optimization approach shown above, the lower level problem maximizes the welfare of the community and optimally distributes the PV generated electricity within the community. This linear problem is replaced by its corresponding KKT conditions to solve the bi-level problem. The lower level KKT formulation is validated by setting the upper-level objective function to a constant (e.g., *F*( *x*) = 1) and ℐ = ℐ _*old*_. With this configuration, the results of the bi-level problem are compared to the solution of the lower level problem without upper-level function, variables, and constraints (which equals the solution of the linear optimization problem based on the model presented in Perger *et al.* ^6^).

**Figure 5.**
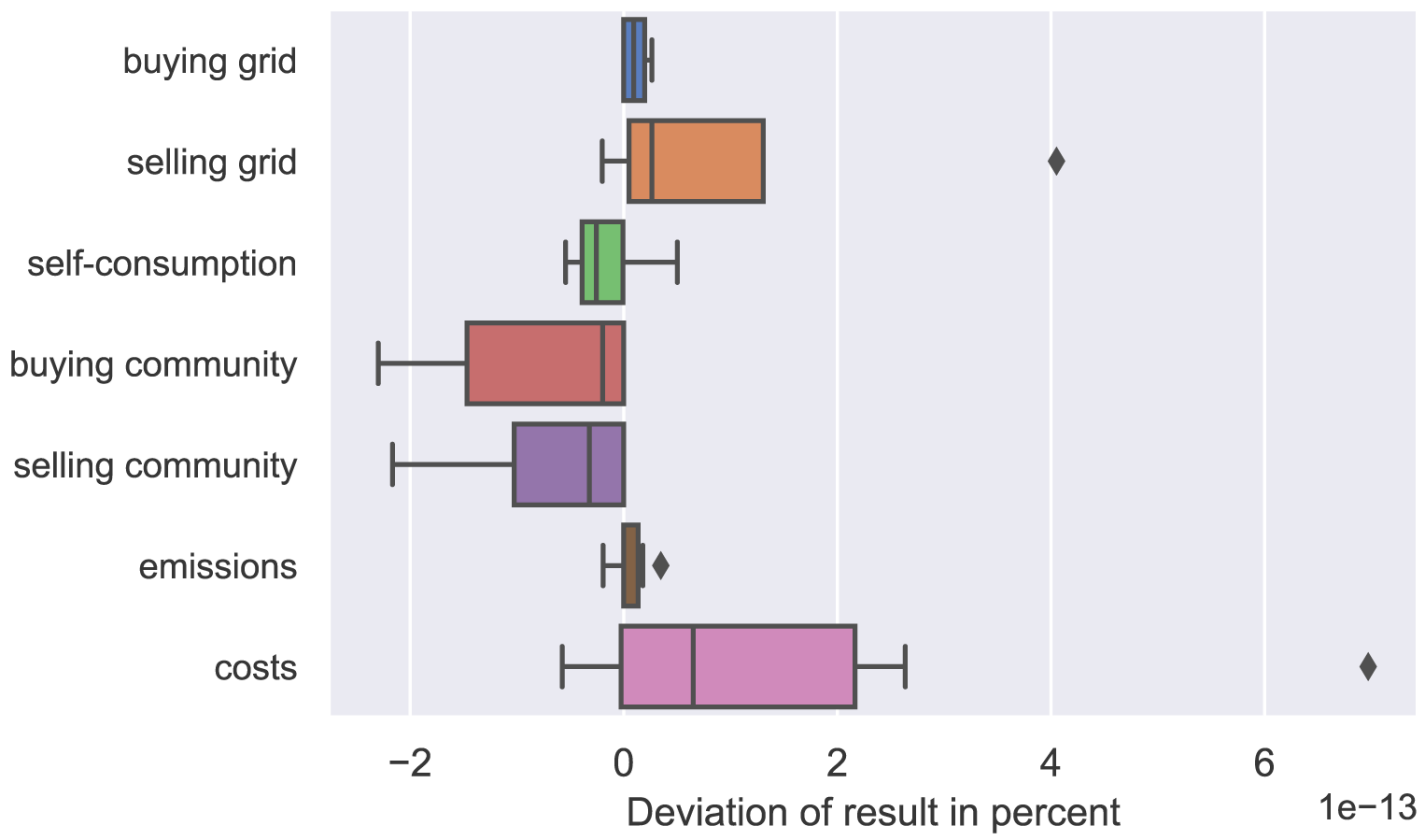
Validation of the Karush-Kuhn-Tucker (KKT) conditions.

The difference of all participants’ annual results (amount of electricity bought and sold, emissions, and costs) is calculated comparing the two solution methods. The box plot in Figure 5 presents the distribution of each category of results. The differences between the two solution methods are negligibly small in the scale of 10 ^–13^ and the KKT formulation of the lower level problem sufficiently substitutes the ordinary LP, which means that the Big-M method is appropriately applied (see Kleinert *et al.* ^61^).

## 4 Results

This section covers the results of the case study described in Section 3.3 under various scenarios, including the original community without extension in Section 4.1, extending the community by adding a new house-hold in Section 4.2.1 and Section 4.2.2, and the comparison of household and business prosumer types in Section 4.3. The different scenarios are compared using fairness indicators in Section 4.4.

### 4.1 Status quo of the original community

**Figure 6.**
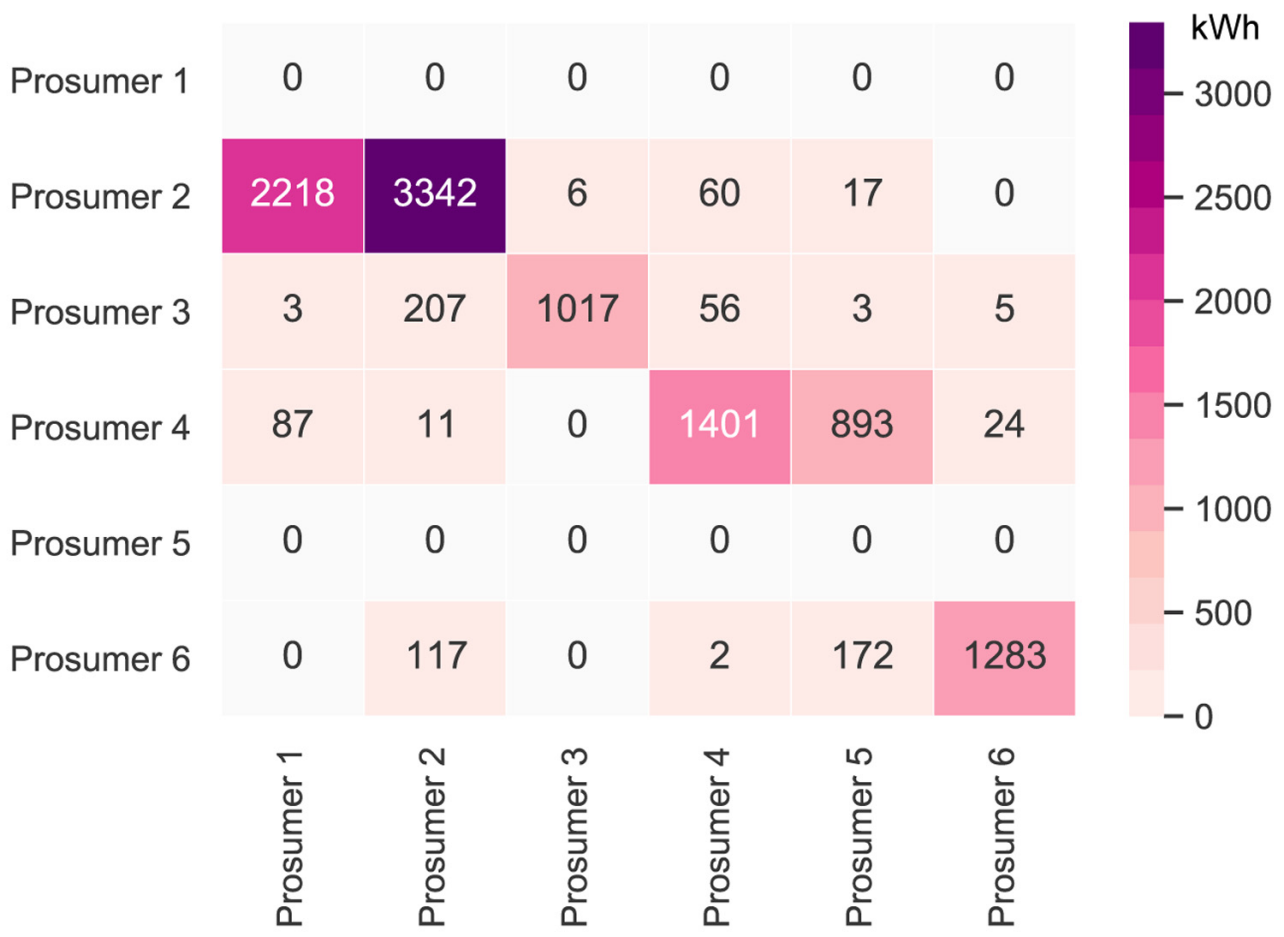
Heatmap of the peer-to-peer electricity trading between the prosumers.

It is first necessary to take a deeper look into the original community’s peer-to-peer trading. The original community consists of six households with consumers and prosumers. The annual results (kilowatt-hours of electricity bought and sold, marginal emissions, and costs) of all members are presented in Table 2. Figure 6 presents the peer-to-peer traded electricity (in kWh */*year) in detail as a heat map; rows represent the amount a prosumer sells to each peer, and columns are the respective purchases.

**Table 2.** Summary of the results of peer-to-peer trading (original community set-up).

Prosumer	1	2	3	4	5	6
Buying grid (kWh)	1140.3	4871.6	1379.3	1080.4	1436.3	854.6
Selling grid (kWh)	0	818.3	1680.0	573.5	0	2286.9
Battery charging (kWh)	0	0	0	870.0	0	0
Battery discharging (kWh)	0	0	0	721.5	0	0
Self-consumption (kWh)	0	3341.5	1016.7	1400.7	0	1282.9
Buying community (kWh)	2308.1	334.6	6.5	117.4	1084.5	29.6
Selling community (kWh)	0	2300.8	274.3	1015.5	0	290.0
Emissions (tCO _2_)	0.6	2.6	0.7	0.6	0.8	0.5
Costs (EUR)	790.0	449.3	154.5	-8.2	527.7	24.0

Compared to all other participants, prosumer 1 buys the most from the community, with the highest share coming from prosumer 2, who is prosumer 1’s closest peer and has a 5kW _peak_ PV system installed. Prosumer 1 does not own a PV system and has the highest willingness-to-pay. Prosumer 3 has the second-highest willingness-to-pay; however, they also have their own PV system installed, and mostly consume their own generation. Prosumer 2 prefers to sell to prosumer 1, with a higher willingness-to-pay than prosumer 3. Prosumer 2 clearly has the highest electricity demand within the community; therefore, the highest annual (marginal) CO _2_ emissions of the community, despite having large PV system capacities installed.

Prosumer 5, who is a consumer only, prefers to buy from their closest peers, prosumers 4 and 6. Prosumer 6 has very low annual electricity costs due to high-self-consumption and being able to sell electricity to other members of the community. Prosumer 4 is the only participant with a BESS and is able to further minimize their electricity costs, achieving negative annual costs.

### 4.2 Results of bi-level optimization of a case study with households

One new prosumer with a household electricity demand profile (prosumer H0) is added to the original community of six households described above. The potential new member is characterized by a willingness-to-pay of 50 EUR */*tCO _2_ (mid-range compared to the other prosumers) and by electrical distances as defined in Figure 4. Minimizing the objective function of the upper-level problem will determine the ideal parameters of the new prosumer. Annual electricity demand might vary between 2000kWh */*year to 8000kWh */*year, and PV capacity between 0kW _peak_ to 5kW _peak_. The variable *n* (number of new prosumers) is set to one; hence, with one potential new prosumer the binary variable *b _i_* automatically equals one (see Eq. (8d)).

***4.2.1 Cost- vs. emission-saving preference of prosumers.*** The first set of results shows two distinct cases; (i) where all members have an emission-saving preference ( *α _i_* = 0), and (ii) where all members have a cost-saving preference ( *α _i_* = 1). A third case (iii) with mixed preferences will be presented in Section 4.2.2.

**Figure 7.**
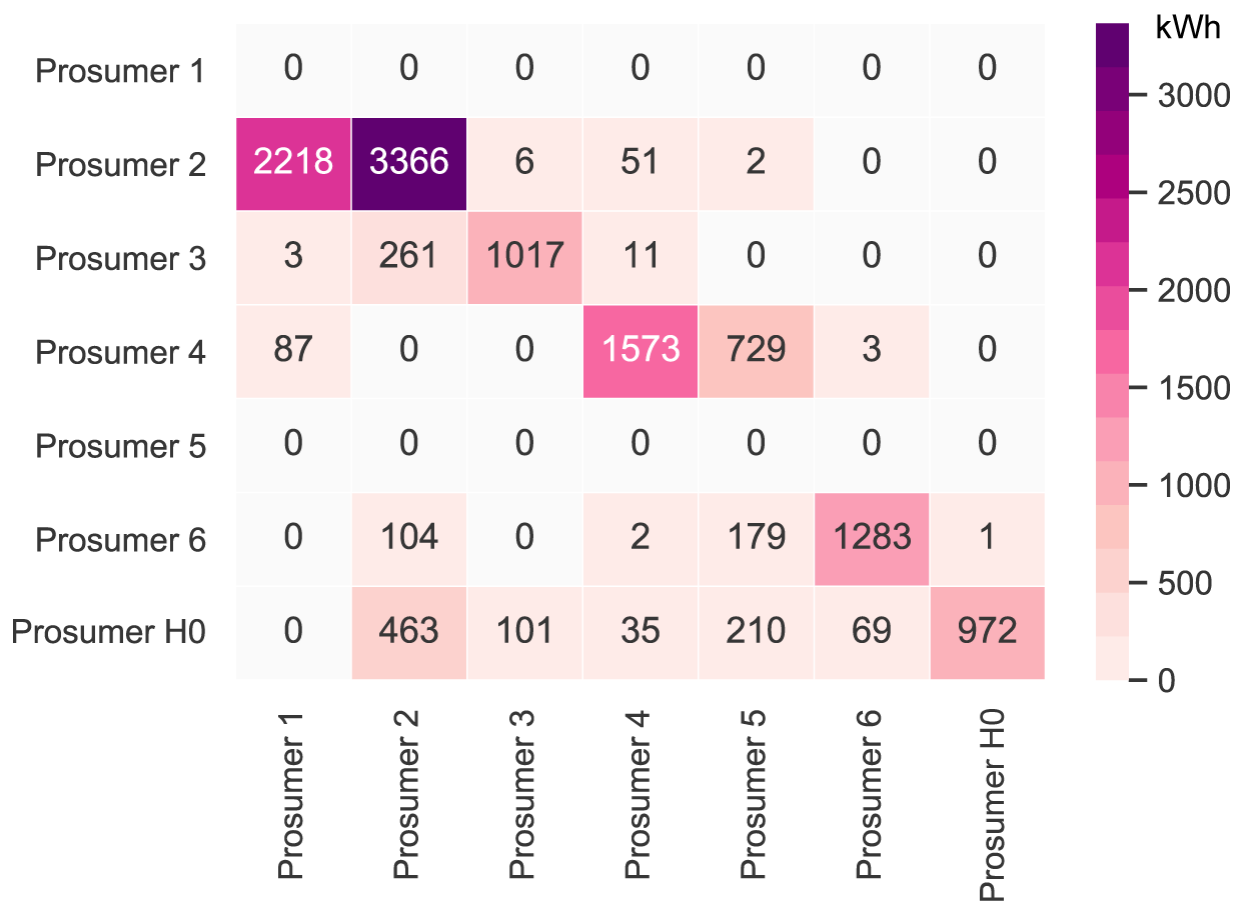
Heatmap of the peer-to-peer electricity trading between the prosumers – all *α _i_* = 0.

**(i) Minimizing emissions** In the first case, it is assumed that all community members care about minimizing their annual emissions, but have no preference regarding cost savings; *α _i_* = 0 is set for all prosumers *i* ∈ ℐ *_old_*. The result of the new prosumer’s PV system size is not surprising. The PV capacity is set to its maximum *PV _new_* = $PV_{new}^{max}$ = 5kW _peak_. At the same time, the optimal electricity demand of the new prosumer is at its minimum *load _new_* = $load_{new}^{min}$ = 2000kWh/year. The new annual peer-to-peer trading values are shown in Figure 7. The annual results (kilowatt-hours of electricity bought and sold, marginal emissions, and costs) of all members are presented in Appendix Table 7.

Cost-wise, the newly added PV capacity can be seen as a competition with other members’ PV systems. Part of the revenue from selling electricity to consumers transfers to the new prosumer instead of old members, whose earnings now decrease. Notably, the annual emissions of all prosumers involved are reduced. Due to the newly added PV capacity, prosumers are able to buy more electricity from the community. The electricity demand of the new prosumer is low, such that there is little competition in consuming PV electricity.

**Figure 8.**
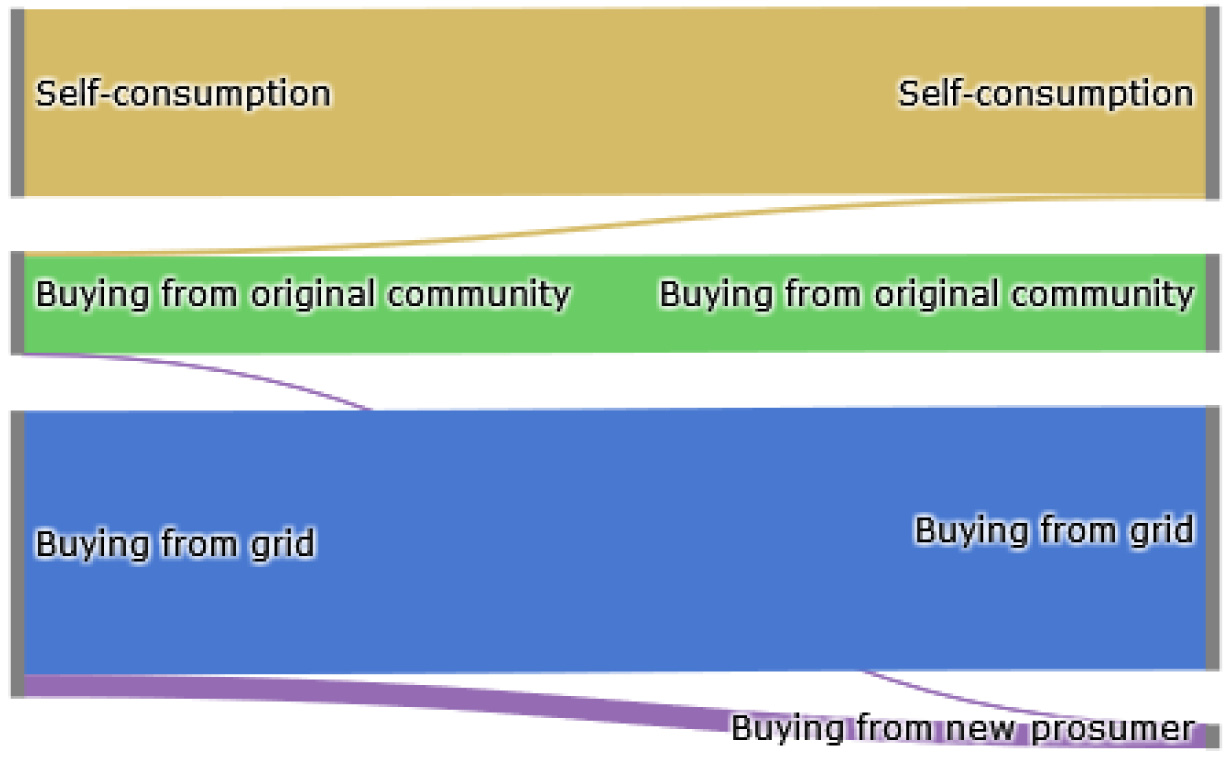
Sankey diagram of the electricity consumption of prosumers.

The Sankey diagram in Figure 8 demonstrates that members of the original community (ℐ *_old_*) cover their electricity demand through self-consumption, buying from other community members or buying from the grid. The left side represents the old community without the new prosumer, and the right side shows the new community. The new prosumer’s PV generation primarily substitutes purchases from the grid, which is desirable if the common goal is to reduce emissions. Prior to adding the new prosumer, community members purchase 10 700kWh from the grid. Adding a new prosumer with a 5kW _peak_ PV system installed, this amount can be reduced by around 8%. Prosumer 4, who has battery storage installed, can also increase their self-consumption.

**Figure 9.**
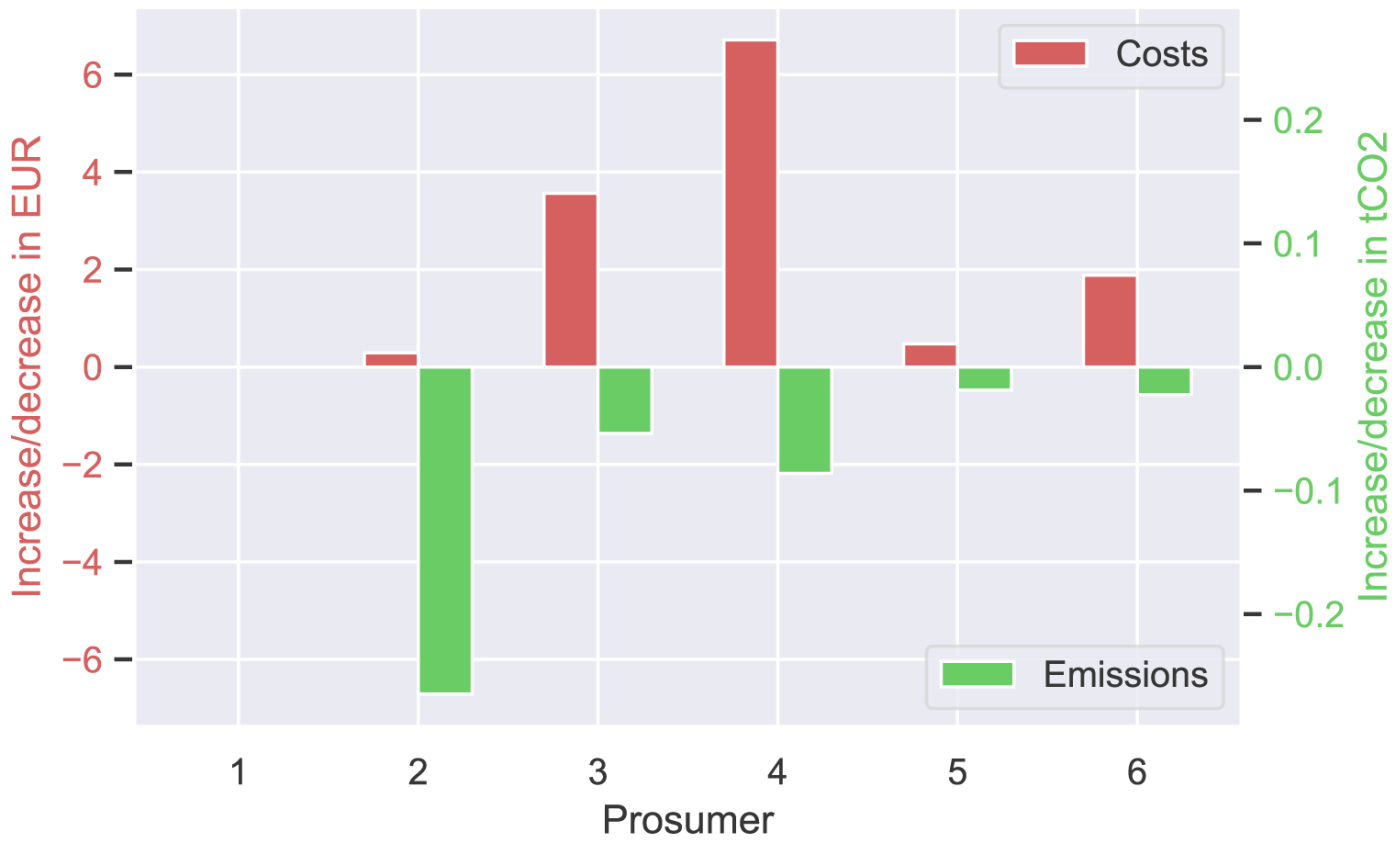
Cost- and emission balances of the prosumer of ℐ _*old*_ – all *α _i_* = 0.

The next Figure 9 presents the annual cost and emission increase (or decrease) of each prosumer of the original community, comparing Eqs. (3) and ( 4). Annual costs (left axis in red) increase slightly by a few EUR for most prosumers, whereas emissions significantly decrease, as desired.

**Figure 10.**
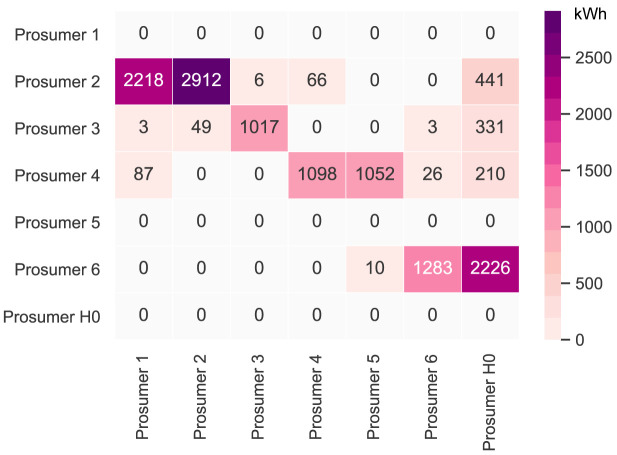
Heatmap of the peer-to-peer electricity trading between the prosumers-all *α _i_* = 1.

**(ii) Minimizing costs** The other distinct case is setting all *α _i_* = 1, indicating that prosumers seek to minimize annual electricity costs. The optimal result of the bi-level problem is a prosumer with the maximum possible annual electricity demand *load _new_* = $load_{new}^{max}$ = 8000kWh */*year. At the same time, the new prosumer’s optimal PV capacity is at its minimum *PV _new_* = $PV_{new}^{min}$ = 0kW _peak_; hence, the new member is a consumer, who buys PV electricity from the community, which generates additional revenue for the other members. The new annual peer-to-peer trading values are shown in Figure 10. The annual results (kilowatt-hours of electricity bought and sold, marginal emissions, and costs) of all members are presented in Appendix Table 8.

**Figure 11.**
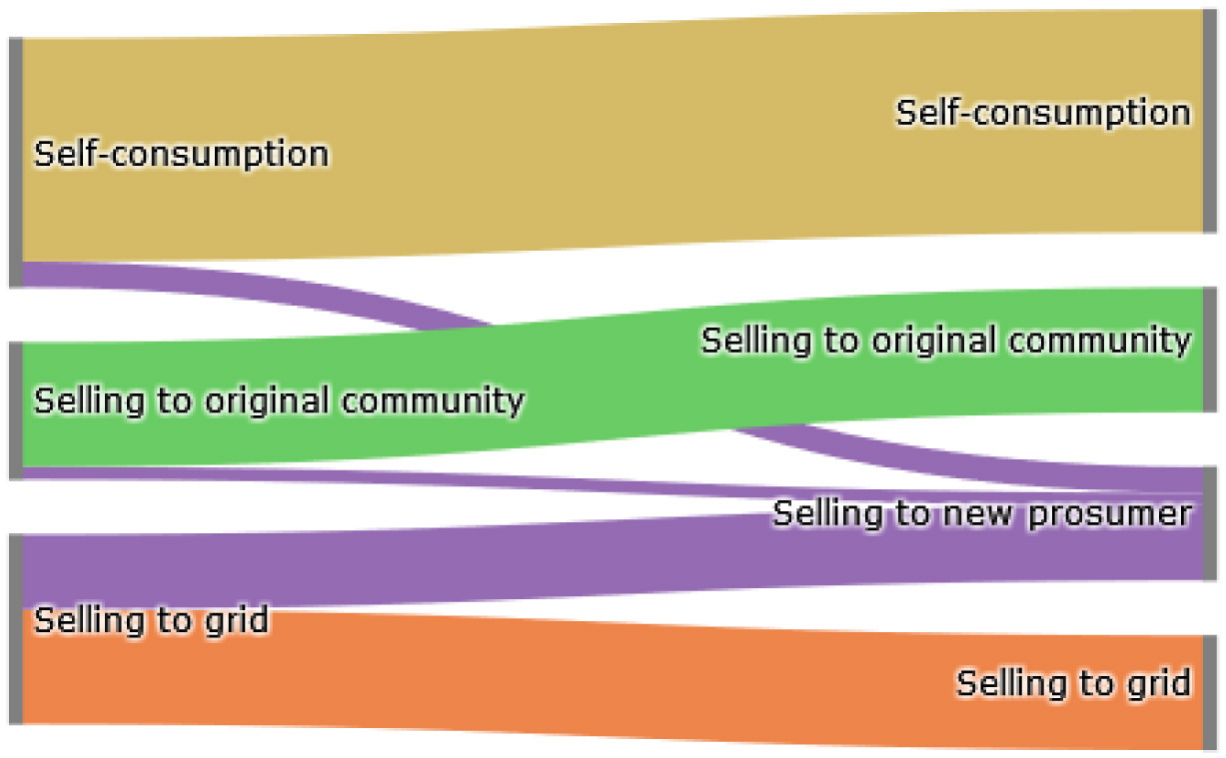
Sankey diagram of the electricity generation of prosumers.

The Sankey diagram in Figure 11 demonstrates that members can increase their income by selling a significant amount of their generation to the new prosumer, which was previously sold to the grid because the new prosumer’s willingness-to-pay is higher than the remuneration for selling PV generation into the grid *wtp _i,new,t_ >* $p_{t}^{G_{out}}$ .

**Figure 12.**
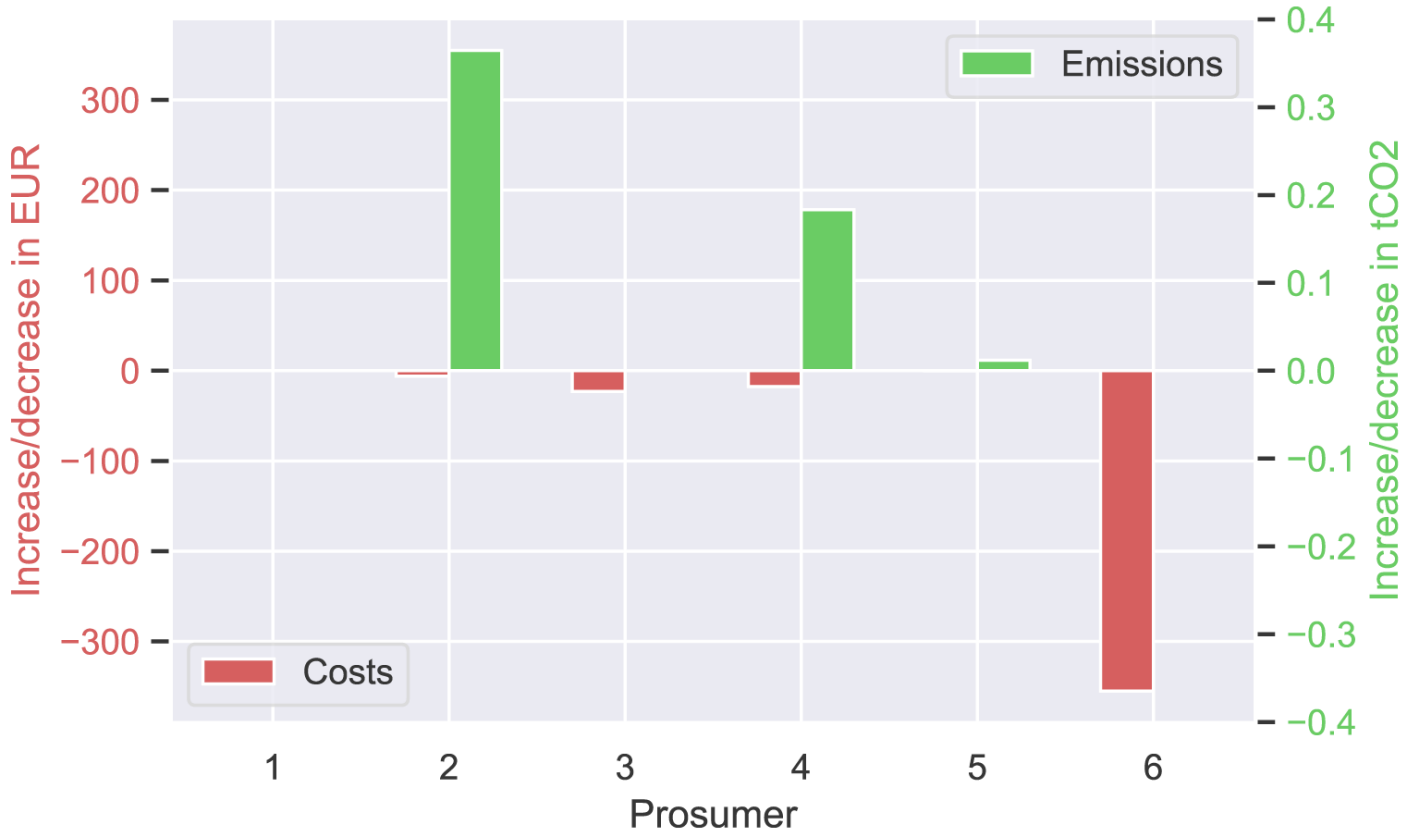
Cost and emission balances of the prosumer of ℐ _*old*_ – all *α _i_* = 1.

In total, about 40% of the community’s surplus PV production is sold to the new prosumer in this scenario, resulting in cost savings for prosumers with PV systems (see Figure 12). This is especially evident for prosumer 6, who is the closest neighbor of the new prosumer. The consumers of the community, prosumers 1 and 5 do not experience major changes. Emission balances offer another interesting result; the lower the willingness-to-pay (e.g., prosumer 2 with *w*_2_ = 0EUR */*tCO _2_), the higher the annual CO _2_ emissions. Prior to adding the new member with a high electricity demand, higher amounts of PV generated electricity remained available for prosumers with low willingness-to-pay, which are now sold to the new member. Prosumer 6, the closest neighbor of the new prosumer, achieves the highest cost decrease.

**Figure 13.**
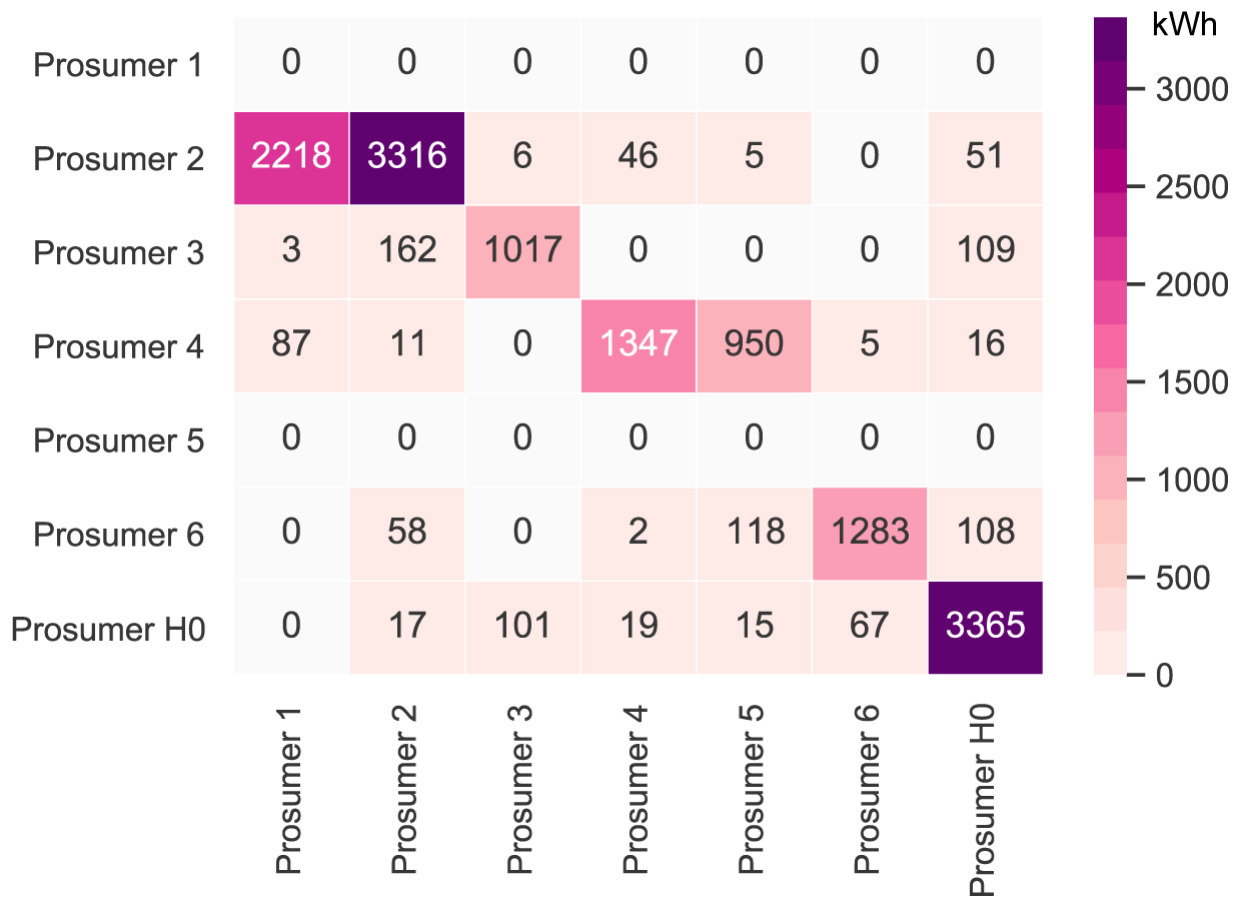
Heatmap of the peer-to-peer electricity trading between the prosumers – mixed *α _i_*.

**Figure 14.**
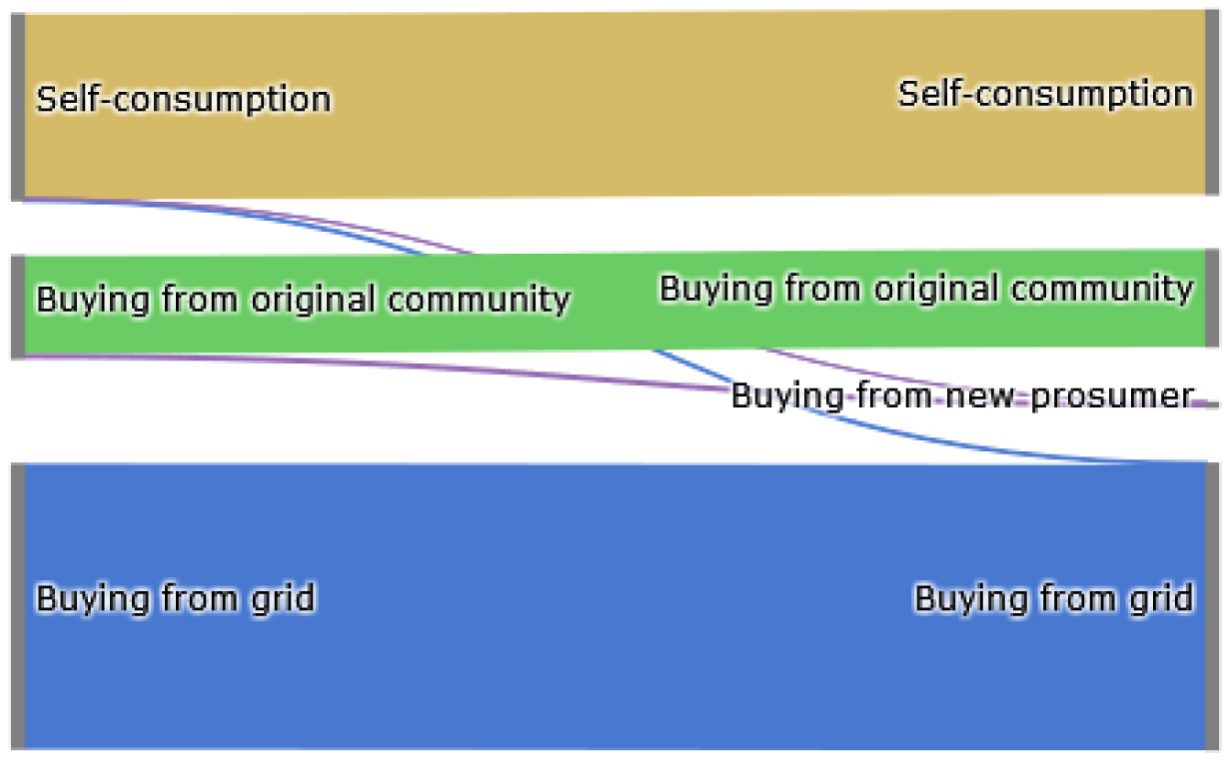
Sankey diagram of the electricity consumption of prosumers.

***4.2.2 Prosumers with mixed emission and cost-saving preferences.*** While the prosumers’ choices of *α _i_* are uniform in both cases (i) and (ii) in Section 4.2.1, this Section introduces non-uniform values of *α _i_*. There is an extremely large number of possible combinations, many of which lead to the same results as either case (i) or (ii). Other combinations lead to different results; for example, [ *α*_1_, *α*_2_, *α*_3_, *α*_4_, *α*_5_, *α*_6_] = [1, 1, 0, 1, 1, 0], which is presented here as case (iii). The optimal parameters of the new prosumer are set by the model to maximum PV capacity and maximum annual electricity demand, *PV _new_* = 5kW _peak_ and *load _new_* = 8000kWh */*year, respectively. The detailed peer-to-peer trading in Figure 13 shows that the new prosumer trades electricity with the other members, but predominantly self-consumes their PV generated electricity due to their own high annual electricity demand. This differs from case (i) in the previous Section, wherein the new prosumer has a low electricity demand and sells larger volumes of electricity to the other members, comparing Figure 14 with Figure 8.

**Figure 15.**
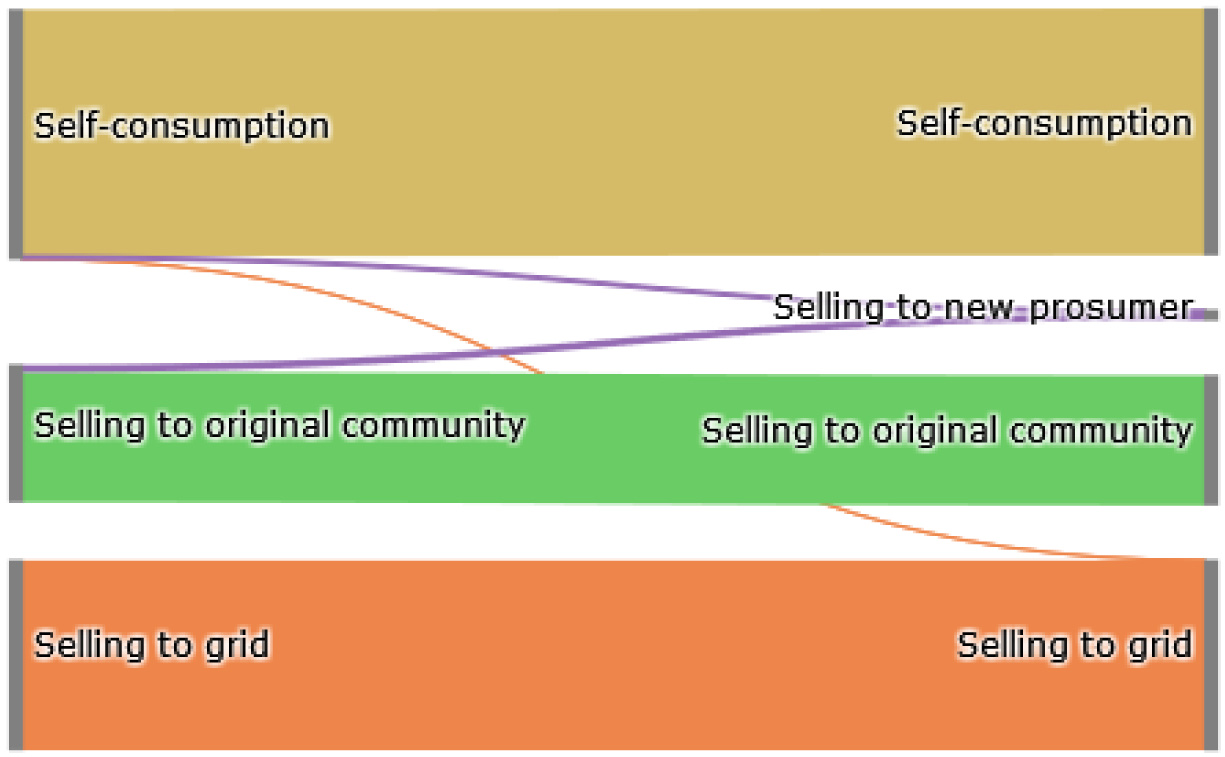
Sankey diagram of the electricity generation of prosumers.

**Figure 16.**
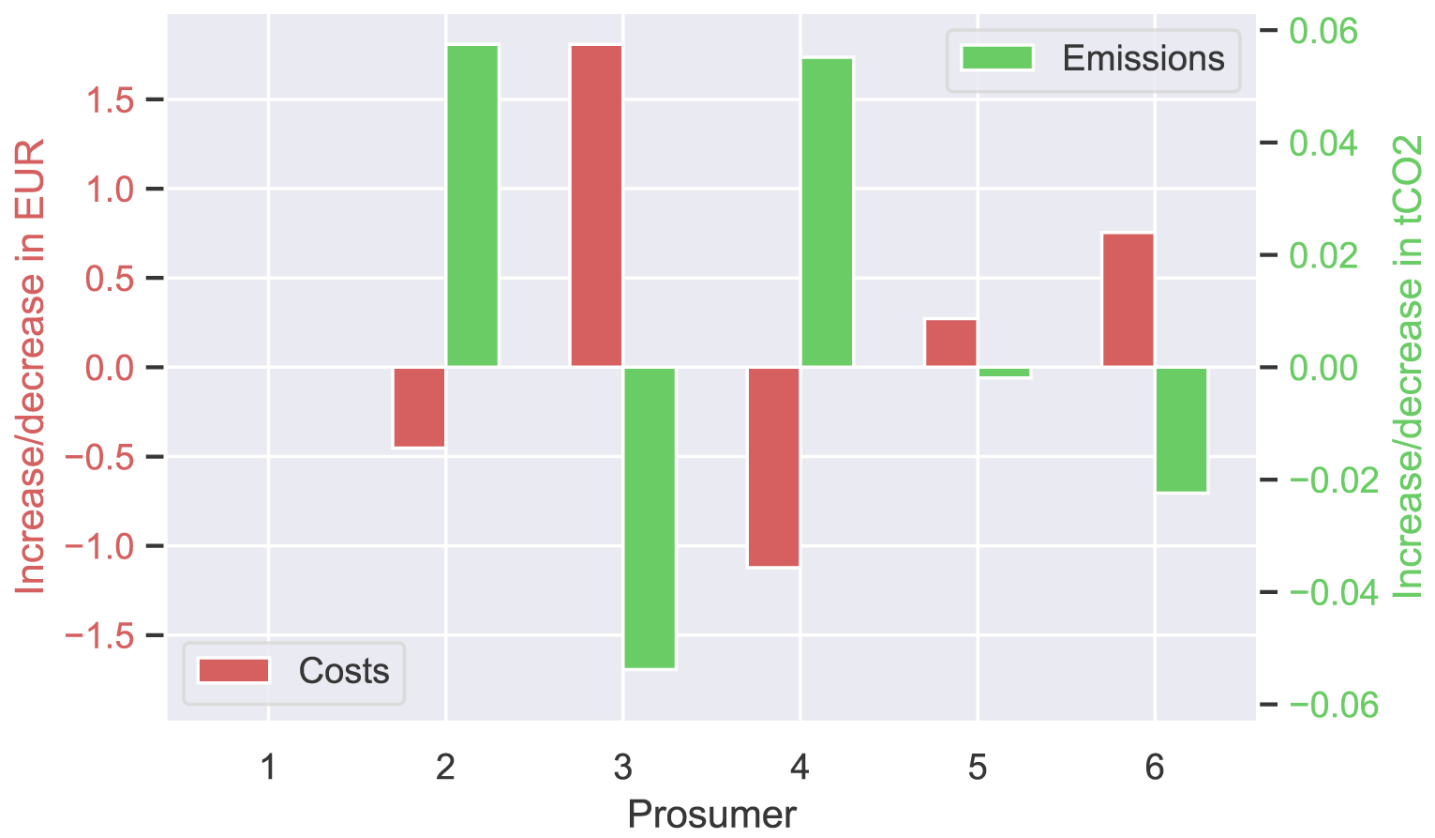
Cost- and emission balances of the prosumer of ℐ _*old*_ – mixed *α _i_*.

Due to the high share of self-consumption in case (iii), the new prosumer buys only small volumes of electricity from the community (see Figure 15). In general, there are less interactions/trades with the community, which is reflected in the annual cost-emission balances as well. Figure 16 shows very small deviations from the previous status quo. Annual emissions decrease for prosumers 3 and 6, which is congruent with their preferences on saving emissions ( *α*_3,6_ = 0). Annual cost differences are negligible (less than 2 EUR per year). The annual results (kilowatt-hours of electricity bought and sold, marginal emissions, and costs) of all members are presented in Appendix Table 9.

### 4.3 Results of bi-level optimization of a case study with households and businesses

Next, another potential new prosumer with the electricity demand profile of a standard business (prosumer G0) is compared to prosumer H0. The results are unchanged when the case study from Section 4.2 is conducted with prosumer G0 instead of H0; therefore, the binary decision variables are actively used in this step and the model is run with two potential new prosumers ℐ _*new*_ = {prosumer H0, prosumer G0} to determine which prosumer type is preferred by the community. There is only one possible choice:


$$\;\sum_{i\in {ℐ}_{new}}b_{i}=1.\;(9)$$

**Figure 17.**
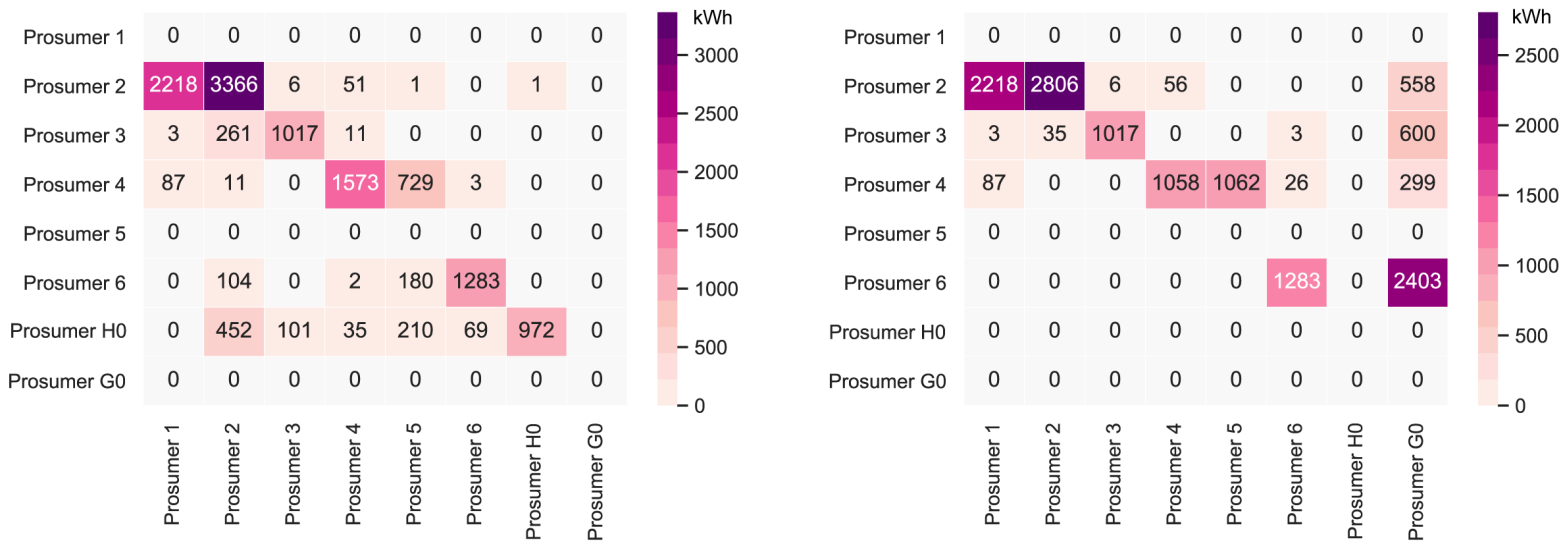
Choosing between prosumer types; *α _i_* = 0 (left) vs. *α _i_* = 1 (right).

We start the analyses by minimizing the individual emissions again, as in case (i). The community prefers the household profile with the same parameters as seen in Section 4.2: *PV _new_* = 5kW _peak_ and *load _new_* = 2000kWh */*year. The annual peer-to-peer trading is shown in Figure 17 (left), wherein the business (prosumer G0) is not part of the community. The other cases, (ii) and (iii), minimizing the prosumers’ costs and mixed preferences elicit a different result. The business is a better match with PV generation profiles than the household (see Figure 22 and Figure 23 in the Appendix) and is, therefore, a better opportunity to sell surplus PV generation to. In case (ii) the business is a consumer only, with an annual electricity demand of 8000kWh (see Figure 17, right). The results are summarized in Table 3.

**Table 3.** Choosing between different prosumer types H0 and G0.

prosumer type	H0	G0
(i) individual emissions	✓	-
(ii) individual costs	-	✓
(iii) mixed *α _i_*	-	✓

### 4.4 Fairness measures

Fairness measures are no introduced to assess the community’s results from a different perspective. Various fairness indicators are used in network technology, which are adapted for peer-to-peer trading, similar to previous work from Moret and Pinson ^16^. The first indicator is Quality of Service (QoS) to measure allocation fairness, referencing Jain’s index (Jain *et al.* ^62^):


$$\;\begin{array}{l} \text{QoS}=\frac{(\sum_{j\in ℐ}q_{j}{)}^{2}}{n\cdot \sum_{j\in ℐ}q_{j}^{2}} \end{array}\;(10)$$

with 


$$\;\begin{array}{l} q_{j}=\sum_{t\in ,i\neq j\in ℐ}\left(q_{i,j,t}^{share}+q_{j,i,t}^{share}\right) \end{array}\;(11)$$

The QoS indicator considers the amount of electricity traded in the community. A QoS of one (100%) indicates perfect fairness, i.e., the trades (buying plus selling) of each member within the community are equally high.

The second indicator is Quality of Experience (QoE):


$$\;\begin{array}{l} \text{QoE}=1–\frac{\sigma }{\sigma_{max}}, \end{array}\;(12)$$

where *σ* is the standard deviation of the perceived electricity costs of the community members (individual electricity costs per unit calculated by dividing the total annual costs by the demand). *σ _max_* is the maximum deviation of perceived electricity costs within the community. A QoE close to one means that there is little deviation in perceived electricity costs (note that the indicator is already slightly distorted by the willingness-to-pay).

The third indicator is minimum-maximum fairness (MinMax), to compare the annual electricity imports of community members from the grid. The MinMax indicator obtains the ratio between the prosumer with smallest amount of electricity imports from the grid and the prosumer with largest imports. A MinMax of one indicates a community of prosumers with similar needs for electricity imports from the grid.


$$\;\begin{array}{l} \text{MinMax}=\frac{\underset{i\in ℐ}{\min}Q_{i}^{G_{in}}}{\underset{i\in ℐ}{\max}Q_{i}^{G_{in}}}, \end{array}\;(13)$$

with the sum of electricity imports from the grid


$$\;\begin{array}{l} Q_{i}^{G_{in}}=\sum_{t\in }q_{i,t}^{G_{in}}. \end{array}\;(14)$$

**Figure 18.**
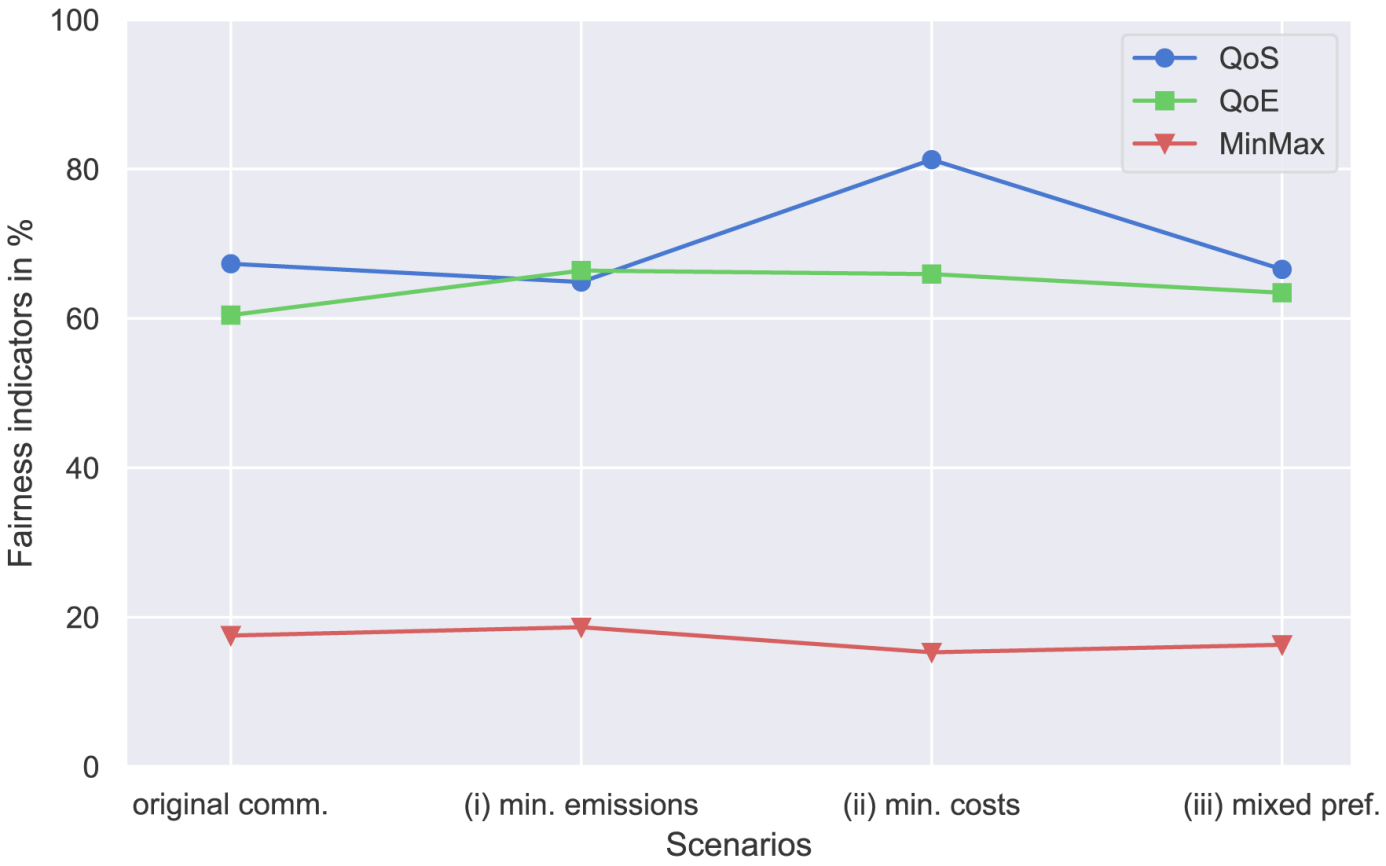
Comparison of different fairness indicators. QoS, Quality of Service; QoE, Quality of Experience; MinMax, minimum-maximum fairness.

These fairness indicators are compared for different sets of results, including the original community ( Section 4.1), case (i) with a preference for minimizing emission and case (ii) with a preference for minimizing costs ( Section 4.2.1), and case (iii) with mixed preferences ( Section 4.2.2). The values are shown in Figure 18.

The QoS indicator is highest in case (ii) with *QoS* = 0.81. In other cases, including the original community, peer-to-peer trading is 65% fair. The new prosumer with a high electricity demand (case (ii)) increases the volumes traded in the community, which are rather fairly distributed within the community, given QoS fairness of more than 80%.

The QoE indicator improves by adding a new prosumer compared to the original community. Interestingly, the indicator is best in case (i). Adding additional PV capacity to the community helps to distribute perceived electricity costs more equally among members. However, the deviation of QoE between the cases is generally rather small.

Adding a new prosumer with high electricity demand slightly decreases MinMax fairness. In general, the MinMax indicator is rather small because prosumers with high demand for electricity naturally have higher volumes of purchases from the grid, especially without BESSs involved. In a community set-up including members with low demand and/or flexibilities, the import needs are rather divergent. Therefore, MinMax fairness is small. 

## 5 Sensitivity analysis

This section presents sensitivity analyses to complete the results of this study. In Section 5.1, differing levels of the new prosumer’s willingness-to-pay are applied to the case study to determine possible changes in the results. In Section 5.2, the distances of the new prosumer to the other members are altered.

### 5.1 Influence of willingness-to-pay

The first set of sensitivity analyses observes the effect of the new prosumer’s willingness-to-pay on the community decision. First, we compare the outputs of the bi-level model for different cases of prosumer preferences *α _i_*, as seen in Section 4.2.1 and Section 4.2.2, varying the new prosumer’s willingness-to-pay. Table 4 presents the results of cases (i)-(iii), where *w _new_* is altered from one side of the spectrum of willingness-to-pay, *w _new_* = 0EUR */*tCO _2_, to the other, *w _new_* = 100EUR */*tCO _2_. There is no noticeable influence of *w _new_* in cases (i) and (ii) (see Table 4). With either all *α _i_* = 0 or *α _i_* = 1, the parameters of the new prosumer, 2000kWh/5kW _peak_ and 8000kWh/0kW _peak_, respectively, are clearly specified by the upper-level cost-emission objective function ( *CE*), regardless the new prosumer’s willingness-to-pay.

In contrast, *w _new_* can be a decisive factor when *α _i_* are mixed. With *w _new_* = 100EUR */*tCO _2_, the new prosumer’s optimal annual electricity demand decreases to 2000kWh, whereas lower willingness-to-pay leads to 8000kWh. Prosumer 6 has a preference to lower emission ( *α*_6_ = 0) in case (iii). When *w _new_ > w*_6_ = 60EUR */*tCO _2_, the peer-to-peer allocation assigns higher volumes of PV generated electricity to the new prosumer instead of prosumer 6, negatively impacting the cost-emission function *CE* and lowering the optimum electricity demand of the new prosumer.

**Table 4.** Influence of the willingness-to-pay on the results (new prosumer is a household). *w _new_* is the individual CO _2_- price of the new prosumer, *load _new_* and *PV _new_* the resulting optimal annual electricity demand and PV capacity of the new prosumer, respectively.

	*w _new_* = 0		*w _new_* = 50		*w _new_* = 100	
	*load _new_*	*PV _new_*	*load _new_*	*PV _new_*	*load _new_*	*PV _new_*
	(kWh)	(kW _peak_)	(kWh)	(kW _peak_)	(kWh)	(kW _peak_)
(i) individual emissions	2000	5	2000	5	2000	5
(ii) individual costs	8000	0	8000	0	8000	0
(iii) mixed preferences	8000	5	8000	5	2000	5

Next, the community decides between two potential new members (similar to Section 4.3) with opposite levels of willingness-to-pay to analyze the influence of the willingness-to-pay on the community’s choice. The first example is two household (H0) prosumers, who are identical except for the willingness-to-pay, *w _H_*_0,0_ = 0 vs. *w _H_*_0,100_ = 100. The community’s choices can be seen in Table 5, columns two and three (highlighted). In cases (i) and (iii), a prosumer with a low willingness-to-pay is preferred, whereas, in case (ii), the community opts for the prosumer with high willingness-to-pay. The two subsequent columns on the right, which compares household (H0) and business (G0) prosumers, repeat this pattern.

**Table 5.** Influence of the willingness-to-pay on the choice of the community. *w _new_* is the individual CO _2_-price of the new prosumers.

prosumer type	H0	H0	H0	G0	G0	H0
*w _i_* in EUR */*tCO _2_	0	100	0	100	0	100
(i) individual emissions	✓	-	✓	-	✓	-
(ii) individual costs	-	✓	-	✓	-	✓
(iii) mixed preferences	✓	-	✓	-	✓	-

An assertion can be drawn from the first set of sensitivity analyses that while willingness-to-pay is not a decisive factor in terms of choosing a new prosumer’s optimal parameters, it is crucial when deciding between two otherwise identical or similar prosumers. This leads to the assumption that willingness-to-pay is a more significant parameter than prosumer type.

### 5.2 Influence of distance criteria

**Figure 19.**
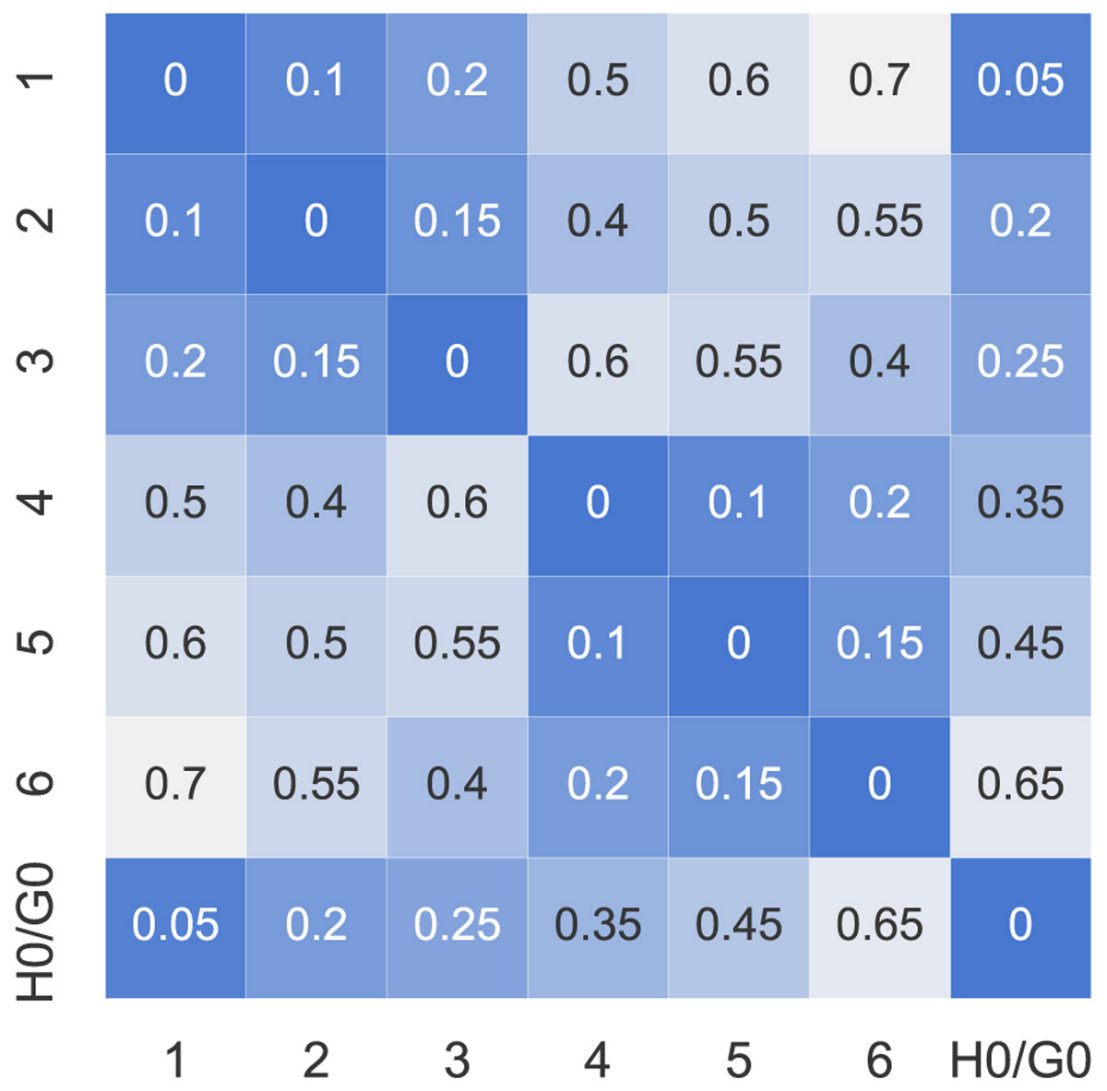
Distance factors *d _ij_* between the members (H0 and G0 represent the new prosumers).

The second type of sensitivity analysis alters the geographical location of the new prosumer with respect to the old community members. The altered distance factors, $\tilde{d}$ , of the new prosumer are mirrored compared to the original configuration, *d*:


$$\;{\tilde{d}}_{new,j}=d_{new,(N+1)-j},\;(15)$$

where *j* are the indices of prosumers in ℐ _*old*_; hence, the new prosumer is (geographically) on the other side of the community. The closest community member is prosumer 1, the furthest is prosumer 6. Note that the distances within the original community remain equal. The new distance factors can be found in Figure 19.

**Figure 20.**
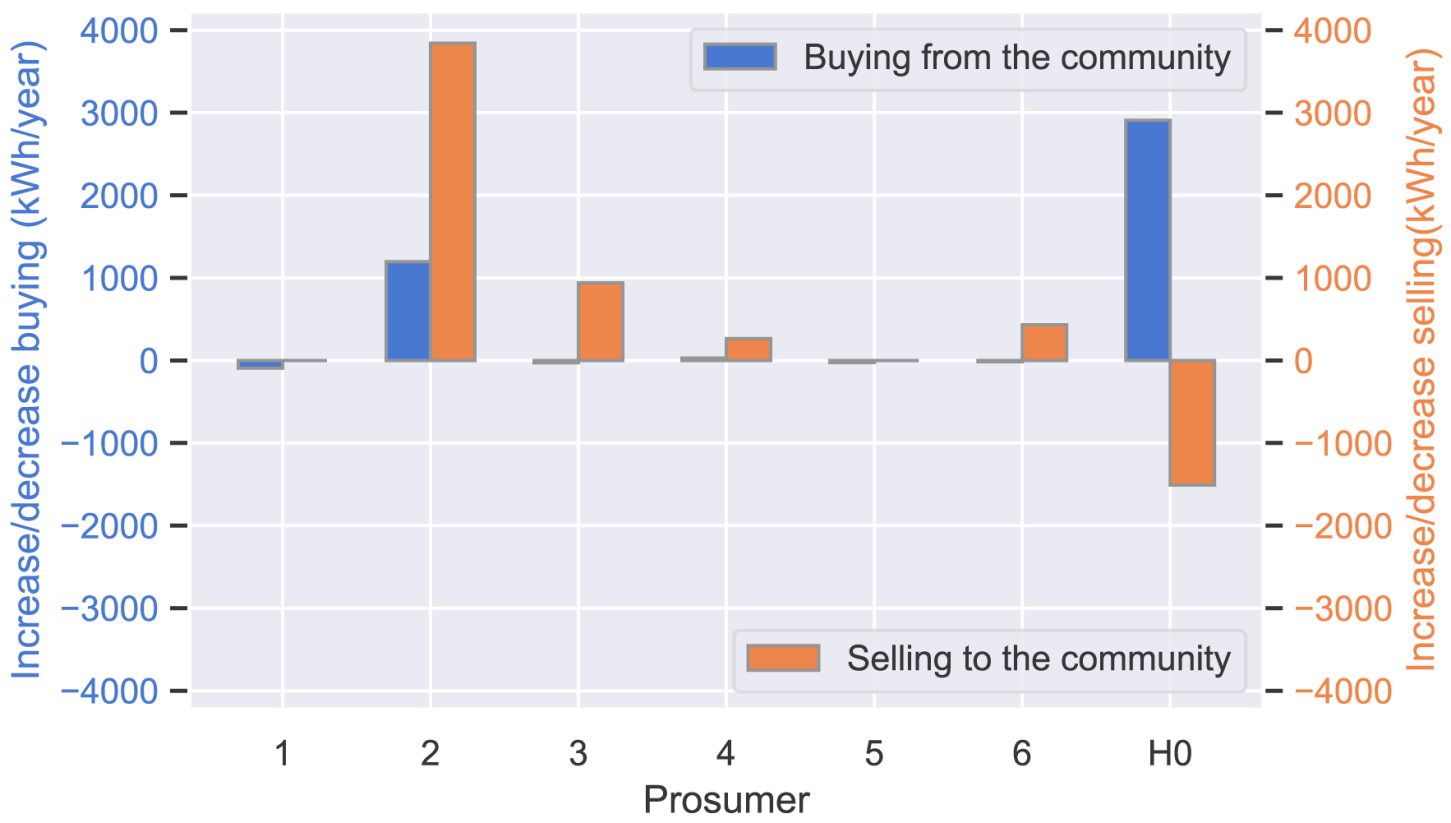
Deviation of buying/selling.

Cases (i)–(iii) are once again analyzed and the new prosumer is a household prosumer type with a willingness-to-pay *w _new_* = 50EUR */*tCO _2_. Deviation from the previous distance set-up is noticeable in case (iii), where the PV capacity changes to zero, whereas the other two cases remain the same, see Table 6. In cases (i) and (ii), the location of the new prosumer does not influence the community’s decision. To analyze the community’s decision in the mixed-preference (case (iii)), Figure 20 compares the prosumer’s volumes of traded electricity in two different scenarios: (a) the optimal output of case (iii) ( *load _new_* = 8000kWh */*year and *PV _new_* = 0kW _peak_) and (b) the non-optimal parameters of the new prosumer ( *load _new_* = 8000kWh */*year and *PV _new_* = 5kW _peak_) in Section 4.2.2, both with new distance factors ${\tilde{d}}_{new,j}.$ The optimal parameters in scenario (a) lead to an increase in purchases from the community and a decrease in sales for the new prosumer (H0) compared to (b). Therefore, the prosumers of ℐ _*old*_ considerably increase sales volumes, particularly prosumer 2 with a cost-saving preference ( *α*_2_ = 1), which compensates for the small decrease in purchases of prosumer 3 and prosumer 6, who have an emission-saving preference ( *α*_3_, *α*_6_ = 0) in case (iii).

**Table 6.** Influence of the willingness-to-pay on the community’s choice. *d _new_* and $\tilde{d}$ *_new_* are the unmodified and modified distance factors, respectively; *load _new_* and *PV _new_* are the the resulting optimal annual electricity demand and PV capacity of the new prosumer, respectively.

	old distances *d _new_*		new distances *d _new_*	
	*load _new_*	*PV _new_*	*load _new_*	*PV _new_*
	(kWh)	(kW _peak_)	(kWh)	(kW _peak_)
(i) ind. emissions	2000	5	2000	5
(ii) ind. costs	8000	0	8000	0
(iii) mixed preferences	8000	5	8000	0

## 6 Conclusions

This work proposes a bi-level optimization model for dynamic participation in energy communities with peer-to-peer trading. The functionality of the model is demonstrated in a small case study and sensitivity analyses.

The model is able to choose the optimal parameters of a new member. This is the first step for gaining useful information on the kind of prosumer (e.g., consumer only or prosumer, high or low PV capacity, level of annual electricity demand, including or excluding BESS (the latter aspect was not shown in this research)) that is preferred by the community. Simultaneously, the model can determine whether the participation of a new member in the community is accepted or rejected; hence, a choice between potential members can be made. In this model, the case study was limited to one new addition to the community; however, it is possible to introduce a portfolio of new members without limiting the number of new members. The model determines the prosumers who are selected and the optimal number of new members at the same time. This is possible because binary variables are bound to each new member that determine acceptance or rejection. The optimal number differs based on the portfolio of members and the needs of the old community.

The community’s choice reflects well the different needs of prosumers. Environment- and profit-oriented preferences are balanced, and there is no bias toward one aspect or the other. Geographical distance and the new prosumer’s willingness-to-pay also influence the decision. If the community members have divergent needs, it is recommended to aim for a diverse set-up of members. There is, of course, also the possibility for the community to define a common goal, such as saving the community’s total emissions. In that case, the community must ensure that new prospective members commit to the same target.

Ultimately, the energy community must be able to attract suitable potential new members to guarantee its performance over the years. If members leave the community and cannot be replaced by new members who restore or improve the status quo, the satisfaction of existing members with the community decreases. In fact, this is a limitation of this work. The selection process is made solely from the perspective of the original community, assuming the availability of potential new prosumers who fit well into the community. Another limitation is that length of the binding contract between participants and community is always one year (and can be extended for another year after expiration); therefore, variations in contract lengths are not included in the decision process.

Future research should include analyses of communities with more diverse participants, such as different settlement patterns (cities or rural areas), community sizes, and other relevant parameters. Another possible future research topic is to study an energy community over a longer period of time (e.g., many years) including members with different contract lengths. Finally, analysis of dynamic participation from the perspective of Distribution System Operators (DSO) and/or community managers should follow.

## A Formulation of the KKT conditions of the lower level problem

### A.1 Lagrangian function

To derive the KKT conditions, the Lagrangian function ℒ must be formulated:


$$\begin{array}{l} ℒ(q_{i,t}^{G_{in}},q_{i,t}^{G_{out}},q_{i,j,t}^{share},q_{i,t}^{B_{in}},q_{i,t}^{B_{out}},SoC_{i,t})\\ =-CW\\ \;+\lambda_{i,t}^{load}(q_{i,t}^{G_{in}}+q_{i,t}^{B_{out}}+\sum_{j\in ℐ}q_{j,i,t}^{share}-q_{i,t}^{load})\\ \;+\lambda_{i,t}^{PV}(q_{i,t}^{G_{out}}+q_{i,t}^{B_{in}}+\sum_{j\in ℐ}q_{i,j,t}^{share}-q_{i,t}^{PV})\\ \;+\lambda_{i,t>t_{0}}^{SoC}(SoC_{i,(t>t_{0})-1}+q_{i,t>t_{0}}^{B_{in}}\cdot \eta^{B}-q_{i,t>t_{0}}^{B_{out}}/\eta^{B}-SoC_{i,t>t_{0}})\\ \;+\lambda_{i,t_{0}}^{SoC}(SoC_{i,t=t_{end}}+q_{i,t_{0}}^{B_{in}}\cdot \eta^{B}-q_{i,t_{0}}^{B_{out}}/\eta^{B}-SoC_{i,t_{0}})\\ \;+\mu_{i,t}^{SoC^{max}}(SoC_{i,t}-SoC_{i}^{max})\\ \;+\mu_{i,t}^{B_{in}^{max}}(q_{i,t}^{B_{in}}-q_{i}^{B^{max}})\\ \;+\mu_{i,t}^{B_{out}^{max}}(q_{i,t}^{B_{out}}-q_{i}^{B^{max}})\\ \;-\beta_{i,t}^{G_{in}}q_{i,t}^{G_{in}}-\beta_{i,t}^{G_{out}}q_{i,t}^{G_{out}}-\beta_{i,j,t}^{share}q_{i,j,t}^{share}-\beta_{i,t}^{B_{in}}q_{i,t}^{B_{in}}-\beta_{i,t}^{B_{out}}q_{i,t}^{B_{out}}-\beta_{i,t}^{SoC}q_{i,t}^{SoC} \end{array}\;(16)$$

### A.2 Formulation of KKT conditions

Stationarity of the Lagrangian function:


$$\;\partial ℒ/\partial q_{i,t}^{G_{in}}=p_{t}^{G_{in}}+\lambda_{i,t}^{load}-\beta_{i,t}^{G_{in}}\;=0\;(17a)$$

$$\;\partial ℒ/\partial q_{i,t}^{G_{out}}=-p_{t}^{G_{out}}+\lambda_{i,t}^{PV}-\beta_{i,t}^{G_{out}}\;=0\;(17b)$$

$$\;\partial ℒ/\partial q_{i,j,t}^{share}=-wtp_{i,j,t}+\lambda_{i,t}^{PV}+\lambda_{j,t}^{load}-\beta_{i,j,t}^{share}\;=0\;(17c)$$

$$\;\partial ℒ/\partial q_{i,t}^{B_{in}}=\lambda_{i,t}^{PV}+\lambda_{i,t}^{SoC}\cdot \eta_{B}+\mu_{i,t}^{B_{in}^{max}}-\beta_{i,t}^{B_{in}}\;=0\;(17d)$$

$$\;\partial ℒ/\partial q_{i,t}^{B_{out}}=\lambda_{i,t}^{load}-\lambda_{i,t}^{SoC}/\eta_{B}+\mu_{i,t}^{B_{out}^{max}}-\beta_{i,t}^{B_{out}}\;=0\;(17e)$$

$$\partial ℒ/\partial SoC_{i,t<t_{end}}=-\lambda_{i,t}^{SoC}+\lambda_{i,t+1}^{SoC}+\mu_{i,t}^{SoC^{max}}-\beta_{i,t}^{SoC}\;=0\;(17f)$$

$$\;\partial ℒ/\partial SoC_{i,t_{end}}=-\lambda_{i,t_{end}}^{SoC}+\lambda_{i,t_{0}}^{SoC}+\mu_{i,t}^{SoC^{max}}-\beta_{i,t}^{SoC}\;=0\;(17g)$$

Substituting $\beta_{i,t}^{G_{in}},\beta_{i,t}^{G_{out}},\beta_{i,j,t}^{share},\beta_{i,t}^{B_{in}},\beta_{i,t}^{B_{out}}\beta_{i,t}^{SoC},$ the stationarity of the Lagrangian function ( 17a)–( 17g) can be formulated with complementarity conditions as well (see Eq.s (18a)–( 18g)). Eq.s (18h)–( 18n) are the complementarity conditions of the lower level problem’s constraints.


$$\;p_{t}^{G_{in}}+\lambda_{i,t}^{load}\geq 0⊥q_{i,t}^{G_{in}}\geq 0\;(18a)$$

$$\;-p_{t}^{Gout}+\lambda_{i,t}^{PV}\geq 0⊥q_{i,t}^{Gout}\geq 0\;(18b)$$

$$\;-wtp_{i,j,t}+\lambda_{i,t}^{PV}+\lambda_{j,t}^{load}\geq 0⊥q_{i,j,t}^{share}\geq 0\;(18c)$$

$$\;\lambda_{i,t}^{PV}\;+\lambda_{i,t}^{SoC}\cdot \eta B+\mu_{i,t}^{B_{in}^{max}}\geq 0⊥q_{i,t}^{B_{in}}\geq 0\;(18d)$$

$$\;\lambda_{i,t}^{load}-\lambda_{i,t}^{SoC}/\eta B+\mu_{i,t}^{B_{out}^{max}}\geq 0⊥q_{i,t}^{B_{out}}\geq 0\;(18e)$$

$$\;-\lambda_{i,t}^{SoC}+\lambda_{i,t+1}^{SoC}+\mu_{i,t}^{SoC^{max}}\geq 0⊥SoC_{i,t<t_{end}}\geq 0\;(18f)$$

$$\;-\lambda_{i,t_{end}}^{SoC}+\lambda_{i,t_{0}}^{SoC}+\mu_{i,t}^{SoC^{max}}\geq 0⊥SoC_{i,t_{end}}\geq 0\;(18g)$$

$$\;q_{i,t}^{G_{in}}+q_{i,t}^{B_{out}}+\sum_{j\in ℐ}q_{j,i,t}^{share}-q_{i,t}^{load}=0⊥\lambda_{i,t}^{load}\;(18h)$$

$$\;q_{i,t}^{G_{out}}+q_{i,t}^{B_{in}}+\sum_{j\in ℐ}q_{i,j,t}^{share}-q_{i,t}^{PV}=0⊥\lambda_{i,t}^{PV}\;(18i)$$

$$\;SoC_{i,t>t_{0}-1}+q_{i,t>t_{0}}^{B_{in}}\cdot \eta^{B}-q_{i,t>t_{0}}^{B_{out}}/\eta^{B}-SoC_{i,t>t_{0}}=0⊥\lambda_{i,t>t_{0}}^{SoC}\;(18j)$$

$$\;SoC_{i,t=t_{end}}+q_{i,t_{0}}^{B_{in}}\cdot \eta^{B}-q_{i,t_{0}}^{B_{out}}/\eta^{B}-SoC_{i,t_{0}}=0⊥\lambda_{i,t_{0}}^{SoC}\;(18k)$$

$$\;0\leq SoC_{i}^{max}-SoC_{i,t}⊥\mu_{i,t}^{SoC^{max}}\geq 0\;(18l)$$

$$\;0\leq q_{i}^{B^{max}}-q_{i,t}^{B_{in}}⊥\mu_{i,t}^{B_{in}^{max}}\geq 0\;(18m)$$

$$\;0\leq q_{i}^{B^{max}}-q_{i,t}^{Bout}⊥\mu_{i,t}^{B_{out}^{max}}\geq 0\;(18n)$$

### A.3 Transformation of complementarity conditions applying the Fortuny-Amat method

The complementarity constraints are reformulated as a mixed-integer program applying the Fortuny-Amat method. The following set of equations shows the transformation of Eq. (18a), the other complementarity constraints, Eq.s (18b)–( 18n), are transformed in the same way.


$$\;p_{t}^{G_{in}}+\lambda_{i,t}^{load}\geq 0\;(19a)$$

$$\;q_{i,t}^{G_{in}}\geq 0\;(19b)$$

$$\;p_{t}^{G_{in}}+\lambda_{i,t}^{load}\leq (1-u_{i,t}^{G_{in}})M_{1}^{G_{in}}\;(19c)$$

$$\;q_{i,t}^{G_{in}}\leq u_{i,t}^{G_{in}}M_{2}^{G_{in}}\;(19d)$$

$$\;u_{i,t}^{G_{in}}\in \left\{0,1\right\}\;(19e)$$

The value of *M* are *M*_1_ = 5000 and *M*_2_ = 2000, which were determined empirically, ensure the feasibility of the model and effectively no numerical problems.

## B Input parameter of the community and the grid

**Figure 21.**
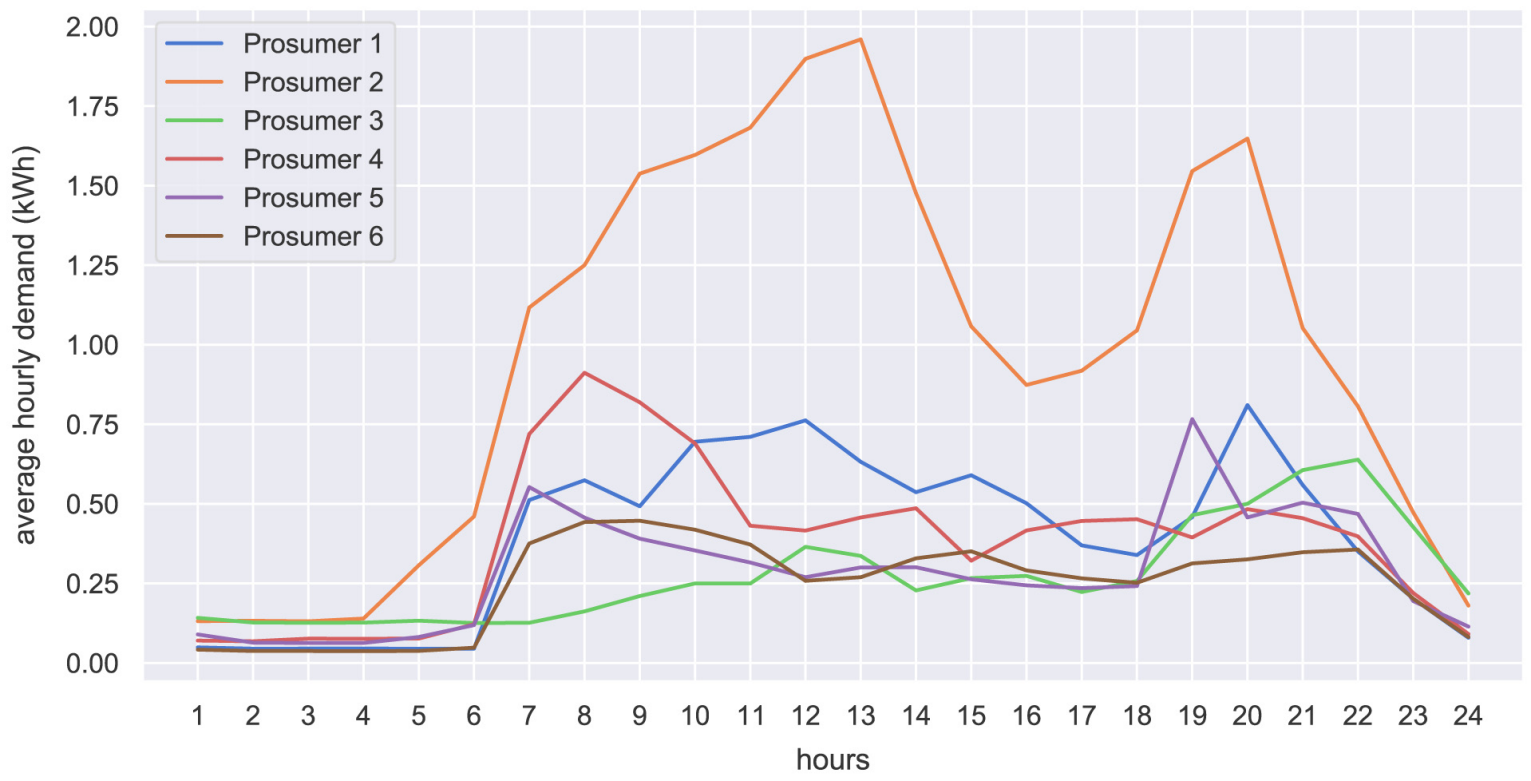
Average hourly electricity demand of prosumers.

**Figure 22.**
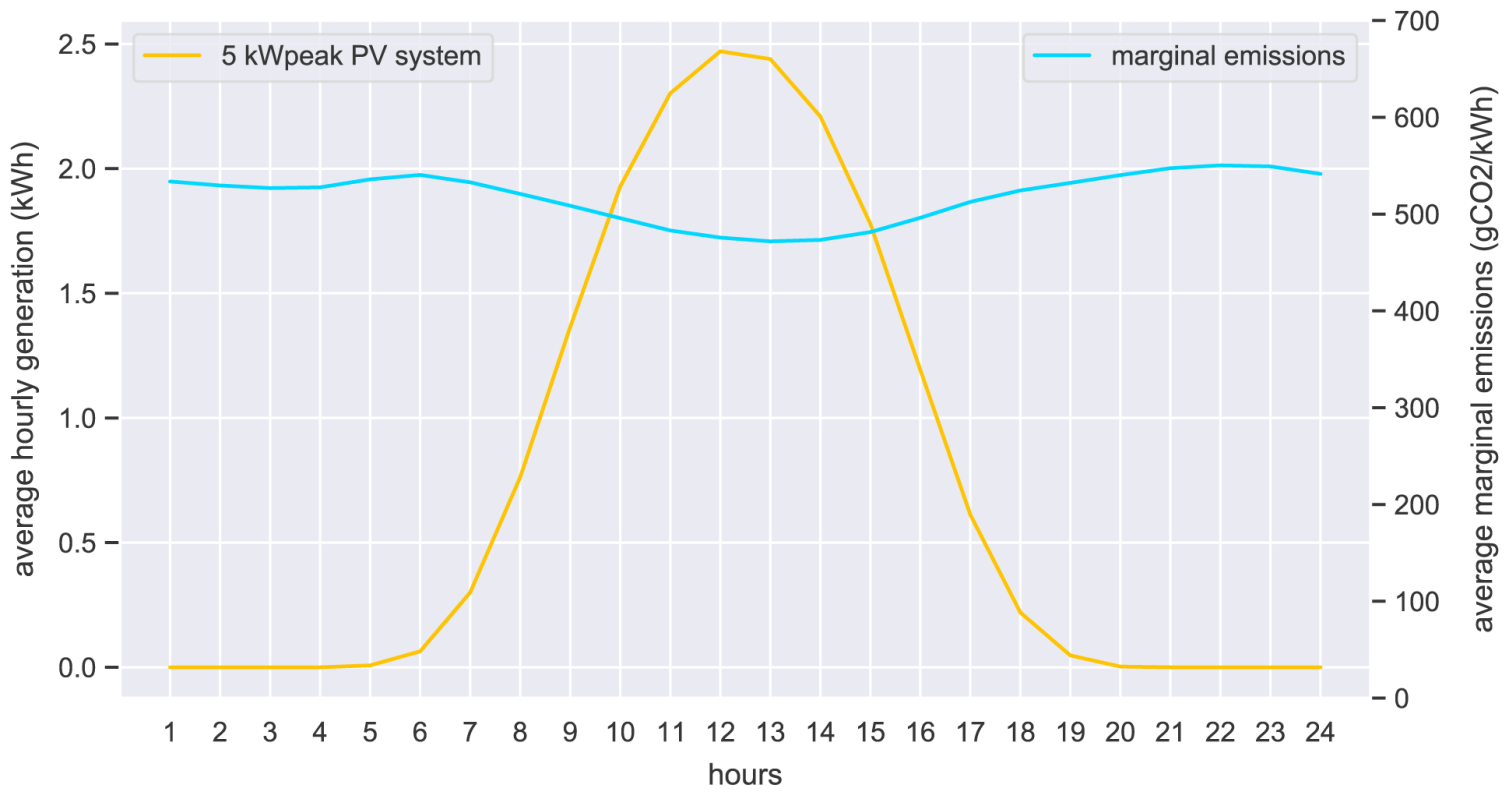
Average hourly electricity PV generation (left) and marginal emissions (right).

**Figure 23.**
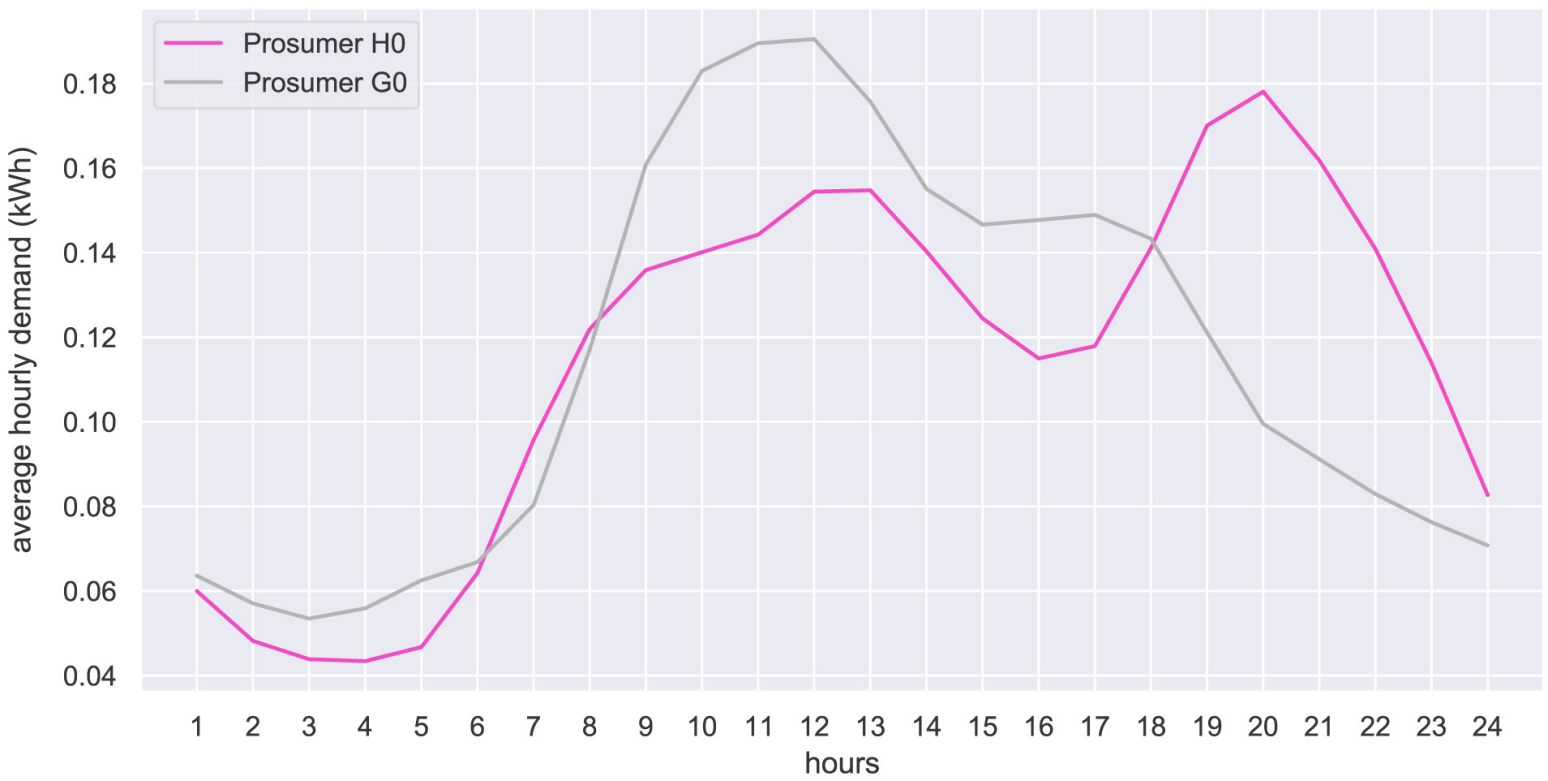
Average hourly electricity demand of new prosumers (normalized to an annual electricity demand of 1000 kWh).

The hourly input data of the case study is presented in the form of hourly average values. The original community prosumers’ electricity demand is shown in Figure 21. The average electricity output values of a 5kW _peak_ PV system is shown in Figure 22 (left axis), together with the marginal emissions from the grid (right axis). Figure 23 shows the standardized load profiles of household H0 and business G0, which are used in the case study to represent the potential new members.

## C Annual results of cases (i)-(iii) in detail

Table 7– Table 9 present the annual results of purchases/sales from/to the grid and the community, self-consumption, battery operation, emissions, and costs for all prosumers 1–6 and prosumer H0. The tables are split into cases (i)–(iii) from Section 4.2.1 and Section 4.2.2.

**Table 7.** Summary of the results of peer-to-peer trading – case (i).

Prosumer	1	2	3	4	5	6	H0
Buying grid (kWh)	1140.3	4354.7	1278.2	917.5	1401	812.6	1027
Selling grid (kWh)	0	818.3	1680	584.6	0	2291.6	4611
Battery charging (kWh)	0	0	0	882.6	0	0	0
Battery discharging (kWh)	0	0	0	731.4	0	0	0
Self-consumption (kWh)	0	3365.6	1016.7	1573.4	0	1282.9	972
Buying community (kWh)	2308.1	827.4	107.6	97.8	1119.8	71.6	0.9
Selling community (kWh)	0	2276.8	274.3	819.2	0	285.4	877.7
Emissions (tCO _2_)	0.6	2.3	0.7	0.5	0.8	0.4	0.6
Costs (EUR)	790	449.5	158.1	-1.4	528.2	25.8	-165

**Table 8.** Summary of the results of peer-to-peer trading – case (ii).

Prosumer	1	2	3	4	5	6	H0
Buying grid (kWh)	1140.3	5587.5	1379.3	1432.6	1459.1	854.6	4792.1
Selling grid (kWh)	0	818.3	1568.3	516.1	0	341.2	0
Battery charging (kWh)	0	0	0	870	0	0	0
Battery discharging (kWh)	0	0	0	723.6	0	0	0
Self-consumption (kWh)	0	2911.6	1016.7	1098.2	0	1282.9	0
Buying community (kWh)	2308.1	48.6	6.5	65.6	1061.7	29.6	3207.9
Selling community (kWh)	0	2730.8	386	1375.4	0	2235.8	0
Emissions (tCO _2_)	0.6	3.0	0.7	0.8	0.8	0.5	2.6
Costs (EUR)	790	443.2	131.6	-25.8	527.6	-331	1663.1

**Table 9.** Summary of the results of peer-to-peer trading – case (iii).

Prosumer	1	2	3	4	5	6	H0
Buying grid (kWh)	1140.3	4983.7	1278.2	1185.8	1432.9	812.6	4351
Selling grid (kWh)	0	818.3	1680	573.5	0	2291.6	2876.6
Battery charging (kWh)	0	0	0	870	0	0	0
Battery discharging (kWh)	0	0	0	720.1	0	0	0
Self-consumption (kWh)	0	3315.6	1016.7	1347.5	0	1282.9	3365
Buying community (kWh)	2308.1	248.4	107.6	66.6	1088	71.6	284
Selling community (kWh)	0	2326.7	274.3	1068.8	0	285.4	219.1
Emissions (tCO _2_)	1	2.7	0.7	0.6	0.8	0.4	2.3
Costs (EUR)	790	448.8	156.3	-9.3	528	24.7	767.4

## Data availability

Zenodo: FRESH:COM Dynamic Participation in Local Energy Communities with Peer-to-Peer Trading. https://doi. org/10.5281/zenodo.5791940 Theresia Perger ^63^.

This project contains the following underlying data:

- *Input_data_alpha.csv* Alpha values ( *α _i_*) of all prosumers.
- *Input_data_distances.csv* Distances ( *d _i, j_*) between the prosumers.
- *Input_data_grid_IAMC.csv* Input data of the grid: electricity prices, marginal emissions (hourly values).
- *Prosumer 1.csv* Input data of Prosumer 1: PV capacity, BESS parameters, willingness-to-pay, electricity demand (hourly values), PV generation (hourly values).
- *Prosumer 2.csv* Input data of Prosumer 2: PV capacity, BESS parameters, willingness-to-pay, electricity demand (hourly values), PV generation (hourly values).
- *Prosumer 3.csv* Input data of Prosumer 3: PV capacity, BESS parameters, willingness-to-pay, electricity demand (hourly values), PV generation (hourly values).
- *Prosumer 4.csv* Input data of Prosumer 4: PV capacity, BESS parameters, willingness-to-pay, electricity demand (hourly values), PV generation (hourly values).
- *Prosumer 5.csv* Input data of Prosumer 5: PV capacity, BESS parameters, willingness-to-pay, electricity demand (hourly values), PV generation (hourly values).
- *Prosumer 6.csv* Input data of Prosumer 6: PV capacity, BESS parameters, willingness-to-pay, electricity demand (hourly values), PV generation (hourly values).
- *Prosumer H0.csv* Input data of Prosumer H0: PV capacity, BESS parameters, willingness-to-pay, electricity demand (hourly values), PV generation (hourly values).
- *Prosumer G0.csv* Input data of Prosumer G0: PV capacity, BESS parameters, willingness-to-pay, electricity demand (hourly values), PV generation (hourly values).

Data are available under the terms of the Creative Commons Attribution 4.0 International license (CC-BY 4.0).

## Software availability

Source code available from: https://github.com/tperger/FRESH-COM

Archived source code at time of publication: https://doi.org/10.5281/zenodo.5796210 Theresia Perger ^64^

License: Apache-2.0

## Footnotes

^1^Community welfare comprises two parts: (i) producer welfare, which considers the community as a whole to maximize producer profits, and (ii) consumer welfare, which considers the individual demand functions (here, willingness-to-pay). Details are explained in Section 3.2.2.

^2^The proposed model calculates optimal BESS sizes as well; however, the focus of this work remains on annual demand and PV system size.

^3^ The synthetic load profiles of 2019 for household (H0 "Haushalt") and business (G0 "Gewerbe allgemein") are used. (See further: https://www.apcs.at/de/clearing/technisches-clearing/lastprofile
