# Supplementary material for: Dynamic participation in local energy communities with peer-to-peer trading
Source: Open Res Eur. 2024 Oct 18;2:5. Originally published 2022 Jan 11. [Version 2] doi: 10.12688/openreseurope.14332.2 (PMC10445879; doi:10.12688/openreseurope.14332.2)
Supplement: Peer review history

# Reviewer report: Reviewer response for version 2

**DOI:** 10.21956/openreseurope.20294.r45291

**Published:** 2025-11-13

**Authors:** Tarek AlSkaif

**Affiliations:** 1 Information Technology Group, Wageningen University and Research, Wageningen, The Netherlands

The authors have addressed most of the reviewer comments and the paper can be accepted for Indexing.

Is the study design appropriate and does the work have academic merit?

Partly

Is the work clearly and accurately presented and does it cite the current literature?

Partly

If applicable, is the statistical analysis and its interpretation appropriate?

Partly

Are all the source data underlying the results available to ensure full reproducibility?

Yes

Are the conclusions drawn adequately supported by the results?

Yes

Are sufficient details of methods and analysis provided to allow replication by others?

Yes

Reviewer Expertise:

Energy informatics and electricity markets

I confirm that I have read this submission and believe that I have an appropriate level of expertise to confirm that it is of an acceptable scientific standard.

# Reviewer report: Reviewer response for version 2

**DOI:** 10.21956/openreseurope.20294.r45289

**Published:** 2024-10-23

**Authors:** Diana Neves

**Affiliations:** 1 IN+ Centre for Innovation, Technology and Policy Research, Universidade de Lisboa, Lisbon, Lisbon, Portugal

The paper is now updated and clearer.

Is the study design appropriate and does the work have academic merit?

Yes

Is the work clearly and accurately presented and does it cite the current literature?

Yes

If applicable, is the statistical analysis and its interpretation appropriate?

Partly

Are all the source data underlying the results available to ensure full reproducibility?

Yes

Are the conclusions drawn adequately supported by the results?

Partly

Are sufficient details of methods and analysis provided to allow replication by others?

Partly

Reviewer Expertise:

Energy systems modelling and analysis

I confirm that I have read this submission and believe that I have an appropriate level of expertise to confirm that it is of an acceptable scientific standard.

# Reviewer report: Reviewer response for version 1

**DOI:** 10.21956/openreseurope.15465.r33501

**Published:** 2023-08-01

**Authors:** Marthe Fogstad Dynge

**Affiliations:** 1 Department of Electric Energy, Norwegian University of Science and Technology, Trondheim, Norway

The article presents a method for existing energy communities to optimally choose new members to join the peer-to-peer energy trading within the community taking individual prosumers’ preferences into account. The work is novel in considering participants entering and leaving the energy community based on the community’s preference. The case studies chosen are extensive and work well in showcasing the proposed methodology’s characteristics. The article is well-written and easy to follow. I also genuinely appreciate the authors’ effort to make the model and data used open-sourced. I only have a few remarks:

 The literature review is extensive and relevant to the conducted research but feels sometimes a bit hasty. The authors mention the objectives of the reviewed literature, rather than their outcomes, thus limiting the value of the state-of-the-art analysis. For example, in 2.2., “In contrast to Germany and the Netherlands, there is a delay in the development and integration of RECs in Southern European countries. Using a survey in Spain and Portugal, Soeiro and Dias aim to understand the motivations of members in energy communities.”. It would be of more interest to the readers to get an insight into the motivations of the Southern European energy communities rather than just knowing that the survey exists.

 “…to optimize energy communities with peer-to-peer trading over the years” is listed as a contribution. I believe that the proposed methodology can be used for this purpose, but the case studies are only over one year and are thus contradicting the contribution statement. I suggest that the contribution statement is reformulated to distinguish between this possible extension and the actual work showcased in this article.

 The choice of comparing adding a household or a business is never reasoned in the article. I assume it is to compare the different load profiles. A sentence or two about this could be added to Section 3.1.1.

 The authors state that clustering mechanisms are used to overcome computational burdens. As the case study only includes six households, I’m curious about the scalability of the proposed method for real-life implementation. Could an indication of the current computational time be included?

 At first glance, I had trouble understanding the heatmaps in Fig. 6, 7, 10 and 13, as it looked like some households were trading electricity with themselves. Could the captions be modified to clarify that the heatmap includes self-consumption?

Is the study design appropriate and does the work have academic merit?

Yes

Is the work clearly and accurately presented and does it cite the current literature?

Yes

If applicable, is the statistical analysis and its interpretation appropriate?

Yes

Are all the source data underlying the results available to ensure full reproducibility?

Yes

Are the conclusions drawn adequately supported by the results?

Yes

Are sufficient details of methods and analysis provided to allow replication by others?

Yes

Reviewer Expertise:

Local energy markets, local energy communities, pricing mechanisms, market models

I confirm that I have read this submission and believe that I have an appropriate level of expertise to confirm that it is of an acceptable scientific standard.

# Response

**Published:** 2024-10-15

**Authors:** Theresia Perger

**Affiliations:** TU Wien, Austria

**We want to thank the reviewer for their time and effort. We appreciate the comments and incorporated them in our updated version. Please see below the comments and our answers. **  

**​​​​​​​Comment:**The article presents a method for existing energy communities to optimally choose new members to join the peer-to-peer energy trading within the community taking individual prosumers’ preferences into account. The work is novel in considering participants entering and leaving the energy community based on the community’s preference. The case studies chosen are extensive and work well in showcasing the proposed methodology’s characteristics. The article is well-written and easy to follow. I also genuinely appreciate the authors’ effort to make the model and data used open-sourced. I only have a few remarks:

 The literature review is extensive and relevant to the conducted research but feels sometimes a bit hasty. The authors mention the objectives of the reviewed literature, rather than their outcomes, thus limiting the value of the state-of-the-art analysis. For example, in 2.2., “In contrast to Germany and the Netherlands, there is a delay in the development and integration of RECs in Southern European countries. Using a survey in Spain and Portugal, Soeiro and Dias aim to understand the motivations of members in energy communities.”. It would be of more interest to the readers to get an insight into the motivations of the Southern European energy communities rather than just knowing that the survey exists. 

**Response:** Thank you for your suggestion to improve the literature review. We added more details to the part where you suggested: “In another work by the same authors, Soeiro and Dias ^40 ^aims to understand the motivations of members in energy communities using a survey in Spain and Portugal.

**Comment:** In said countries, citizens are willing to participate in energy communities if it benefits their local community and the environment.” and to another part where further explanations are useful: “Fair revenue sharing and exit clauses are examined in Fioriti et al. ^43 ^, to identify the optimal sizing of energy communities. Exit clauses, where a member leaving the community has to pay a time-decreasing fee, are introduced to ensure that the remaining members of the community do not experience any economic disadvantages. “   “…to optimize energy communities with peer-to-peer trading over the years” is listed as a contribution. I believe that the proposed methodology can be used for this purpose, but the case studies are only over one year and are thus contradicting the contribution statement. I suggest that the contribution statement is reformulated to distinguish between this possible extension and the actual work showcased in this article.  

**Response: **This is correct. The text is reformulated to clarify the contribution: “ A method is developed based on the peer-to-peer allocation mechanism presented in 6 to optimize energy communities with peer-to-peer trading, including the annual entry and exit of members.”  

**​​​​​​​Comment: **The choice of comparing adding a household or a business is never reasoned in the article. I assume it is to compare the different load profiles. A sentence or two about this could be added to Section 3.1.1.  

**Response: **You are right with your assumption. We added two sentences to better explain our choice.

**​​​​​​​Comment: **“Different consumer types are considered to show a wider variety of load profile types in the analysis. Along with households, small businesses are also a key target group for RECs.”   The authors state that clustering mechanisms are used to overcome computational burdens. As the case study only includes six households, I’m curious about the scalability of the proposed method for real-life implementation. Could an indication of the current computational time be included?   The case study could be computed in less than two minutes on a standard laptop.    At first glance, I had trouble understanding the heatmaps in Fig. 6, 7, 10 and 13, as it looked like some households were trading electricity with themselves. Could the captions be modified to clarify that the heatmap includes self-consumption?  

**Response: **Thank you for your suggestion. We added an explanation to the text where the first heatmap occurs. In addition, we added this information to each of the captions of the heatmaps, as you suggested.

# Reviewer report: Reviewer response for version 1

**DOI:** 10.21956/openreseurope.15465.r33503

**Published:** 2023-07-24

**Authors:** Diana Neves

**Affiliations:** 1 IN+ Centre for Innovation, Technology and Policy Research, Universidade de Lisboa, Lisbon, Lisbon, Portugal

**General**: This study is innovative in the sense that includes the members entries and exits dynamics when analysing the EC performance, under a P2P trading market.

**LR**: it gives a succinct LR from different points of view.

**Methods**: it is not given any rationale on the PV sizing: why does it vary between 0 and 5kW? Also I didn’t see any tilt info (just azimuth).

**Results & conclusions**: it is a bit difficult to follow the scenarios that are being modelled in each result table/graph. There are a lot of results to analysed and without a clear detail on which scenarios were drawn, it is difficult to follow. Though, the assessment parameters are very well chosen.

 I have some doubts with analysing the distances, since there was no formulation about power losses – so I can’t see how you derive conclusions with old and new distances regarding power load. Also, it would be interesting to see the price formation results on the P2P market instead of just the savings on prosumers. To better understand if the main advantage on the savings comes from the P2P or from i.e. self-consumption. Finally, some overall community results would also be interesting to analyse (not only the individual prosumer results), as also some comparison with existent literature to understand if the addition of the entries and exists from the EC modifies greatly the EC results that are already discussed in other literature.

Is the study design appropriate and does the work have academic merit?

Yes

Is the work clearly and accurately presented and does it cite the current literature?

Yes

If applicable, is the statistical analysis and its interpretation appropriate?

Partly

Are all the source data underlying the results available to ensure full reproducibility?

Yes

Are the conclusions drawn adequately supported by the results?

Partly

Are sufficient details of methods and analysis provided to allow replication by others?

Partly

Reviewer Expertise:

Energy systems modelling and analysis

I confirm that I have read this submission and believe that I have an appropriate level of expertise to confirm that it is of an acceptable scientific standard, however I have significant reservations, as outlined above.

# Response

**Published:** 2024-10-15

**Authors:** Theresia Perger

**Affiliations:** TU Wien, Austria

We want to thank the reviewer for their time and effort. We appreciate the comments and incorporated them in our updated version. Please see below the comments and our answers.

**Reviewer comment-**

**General: **This study is innovative in the sense that includes the members entries and exits dynamics when analyzing the EC performance, under a P2P trading market.

**LR:** It gives a succinct LR from different points of view.

**Methods:** It is not given any rationale on the PV sizing: why does it vary between 0 and 5kW? Also I didn’t see any tilt info (just azimuth).

**Author response-** Tilt information was added in Section 3.3.2 in two occasions: “Various PV system sizes and orientations are considered in the analyses presented in this paper, with the tilt uniformly set at 35°.”  and “ qi,tPV is the generation profile of a 1kW _peak_ PV system facing South with a tilt of 35°; hence, the decision variable PV _i_ is a factor that upscales the PV system size. The possible range is between 0 – 5 kW _peak_ from no PV system to a medium sized one.“ The last sentence is also an explanation for the PV system size range. 

**Reviewer comment-**

**Results & conclusions:** It is a bit difficult to follow the scenarios that are being modelled in each result table/graph. There are a lot of results to analyze and without a clear detail on which scenarios were drawn, it is difficult to follow. Though, the assessment parameters are very well chosen.

**Author response-** Thank you for your comment. To make the results more comprehensible for the readers, we use labels for the scenarios (case (i)-case (iii)), describe them and add them to the captions.

**Reviewer comment- **I have some doubts with analyzing the distances, since there was no formulation about power losses – so I can’t see how you derive conclusions with old and new distances regarding power load. 

**Author response-** We added a footnote to Section 3.2.1 to explain more details about the distance factor. “Network topologies must be taken into account when calculating electrical distances. The distance factor is specified in this paper on a scale from zero to one. Zero corresponds to the shortest distance, i.e. the distance from the prosumer to itself. One corresponds to the longest electrical distance in this part of the grid. Power losses are not taken into account directly. The grid losses are charged to the consumers via the grid tariffs in their electricity bill.”  Regarding your question about old and new distances. The new member is at a different location in Section 5.2, hence, the distance factors are different from the rest of the study. In real life this means a different house joins the energy community. 

**Reviewer comment- ** Also, it would be interesting to see the price formation results on the P2P market instead of just the savings on prosumers. To better understand if the main advantage on the savings comes from the P2P or from i.e. self-consumption. 

**Author response- **Thank you for this suggestion. We added a few more results (in text form) based on your suggestion, regarding the cost savings of the community as a whole vs. savings resulting from self-consumption to show that there is indeed an advantage coming from peer-to-peer trading. See e.g. in Section 4.2.1 “Prior to adding the new member with a high electricity demand, higher amounts of PV generated electricity remained available for prosumers with low willingness-to-pay, which are now sold to the new member. Therefore, the savings from self-consumption for the members of the original community (ℐ _old_ ) decrease in total by 146.5 EUR. This minus is compensated by additional income from peer-to-peer trading with the new prosumer. This leads to overall cost savings of 401.6 EUR. Prosumer 6, the closest neighbor of the new prosumer, achieves the highest cost decrease.”

**Reviewer comment- **Finally, some overall community results would also be interesting to analyze (not only the individual prosumer results), as also some comparison with existent literature to understand if the addition of the entries and exists from the EC modifies greatly the EC results that are already discussed in other literature.

**Author response- **Thank you for this comment. We now show results for the community as a whole, see comment above. Regarding other literature: We believe that it is best to understand how the addition of the entries and exits from the energy community modifies the results when we compare the different results of our own community set-up rather than comparing them with existing literature, since there are different conditions, assumptions, etc.

# Reviewer report: Reviewer response for version 1

**DOI:** 10.21956/openreseurope.15465.r33495

**Published:** 2023-07-19

**Authors:** Tarek AlSkaif

**Affiliations:** 1 Information Technology Group, Wageningen University and Research, Wageningen, The Netherlands

This work deals with peer-to-peer electricity trading in local energy markets. It uses bi-level optimization model to determine the parameters of market participants who join the energy community (prosumers/consumers) and considers their preferences (e.g., environmental, economic, or mixed). The subject matter is interesting and the problem is formulated correctly. Some remarks:

1. There have been extensive literature on this topic using centralized/decentralized optimization models and considering prosumers/consumers preferences, even for specific countries (Netherlands, UK, etc). Hence, not all the contributions in Section 2.3 could be considered new. The work needs to clearly discuss recent studies in this domain and contrasts their work and their contribution.
2. Although the lack of theoretical novelty could be seen as a concern, from the reviewer opinion a paper with clear conceptual novelty and thorough/accurate execution of ideas using real data and that informs regulators and stakeholders could offset to a large extent the lack of clear mathematical/theoretical novelty and therefore be of interest. The authors could work on filling this gap to balance their work.
3. Following on the previous comment: the model is tested for 6 prosumers only. This is in my opinion the weakest point in the article. Testing the model on 6 households only is not sufficient to neither drive energy/policy related conclusions nor to test the scalability and performance of the optimization model. More quantitative analysis is needed. The reviewer appreciates the transparency of the author in publishing the data they used for reproducibility.
4. The paper should better define and justify the hypothesis and simplifications, such as that the grid model and power losses in the network appear to be neglected and the prediction of demand and supply is considered deterministic in the numerical analysis and optimization model.

Is the study design appropriate and does the work have academic merit?

Partly

Is the work clearly and accurately presented and does it cite the current literature?

Partly

If applicable, is the statistical analysis and its interpretation appropriate?

Partly

Are all the source data underlying the results available to ensure full reproducibility?

Yes

Are the conclusions drawn adequately supported by the results?

Yes

Are sufficient details of methods and analysis provided to allow replication by others?

Yes

Reviewer Expertise:

Energy informatics and electricity markets

I confirm that I have read this submission and believe that I have an appropriate level of expertise to confirm that it is of an acceptable scientific standard, however I have significant reservations, as outlined above.

# Response

**Published:** 2024-10-15

**Authors:** Theresia Perger

**Affiliations:** TU Wien, Austria

**We want to thank the reviewer for their time and effort. We appreciate the comments and incorporated them in our updated version. Please see below the comments and our answers. ** This work deals with peer-to-peer electricity trading in local energy markets. It uses bi-level optimization model to determine the parameters of market participants who join the energy community (prosumers/consumers) and considers their preferences (e.g., environmental, economic, or mixed). The subject matter is interesting and the problem is formulated correctly. Some remarks:

1. **There have been extensive literature on this topic using centralized/decentralized optimization models and considering prosumers/consumers preferences, even for specific countries (Netherlands, UK, etc). Hence, not all the contributions in Section 2.3 could be considered new. The work needs to clearly discuss recent studies in this domain and contrasts their work and their contribution.**

As you suggested, we improved the quality of the literature review of the paper by adding the following recent references from 2023 and 2024. https://doi.org/10.1016/j.segan.2023.101059 https://doi.org/10.1016/j.scs.2023.104495 https://doi.org/10.1016/j.apenergy.2024.123933 https://doi.org/10.1016/j.segan.2023.101075 https://doi.org/10.1016/j.energy.2024.132418 https://doi.org/10.1016/j.renene.2023.04.094 https://doi.org/10.1016/j.rser.2022.113055 https://doi.org/10.1016/j.ijepes.2024.110041 

1. **Although the lack of theoretical novelty could be seen as a concern, from the reviewer opinion a paper with clear conceptual novelty and thorough/accurate execution of ideas using real data and that informs regulators and stakeholders could offset to a large extent the lack of clear mathematical/theoretical novelty and therefore be of interest. The authors could work on filling this gap to balance their work.**

Thank you for your suggestion. We believe the method does present a novel approach, as integrating a peer-to-peer trading allocation problem with the selection process for new members in an energy community into a bi-level framework is a unique contribution. While the case study is an artificial scenario, the results are derived from real data. Emission levels, electricity prices, and PV generation profiles are real and specific to Austria during the relevant period, ensuring that the community set-up reflects a real-world energy community, which could exist in practice.

1. **Following on the previous comment: the model is tested for 6 prosumers only. This is in my opinion the weakest point in the article. Testing the model on 6 households only is not sufficient to neither drive energy/policy related conclusions nor to test the scalability and performance of the optimization model. More quantitative analysis is needed. The reviewer appreciates the transparency of the author in publishing the data they used for reproducibility.**

Thank you for this comment and your appreciation of data transparency and reproducibility. The choice of a small energy community to test the model is not unrealistic, as there are also many small energy communities in real life. The scalability was tested in our follow-up research, where we extended the model to a more complex, stochastic selection process with up to 20 members, see https://doi.org/10.1007/s00502-022-01069-2.     

1. **The paper should better define and justify the hypothesis and simplifications, such as that the grid model and power losses in the network appear to be neglected and the prediction of demand and supply is considered deterministic in the numerical analysis and optimization model.**

Thank you for this suggestion to improve the paper. We added an additional Section “3.5 Limitations of the modeling approach The bi-level modeling approach and the study presented in this work contain the following limitations. Firstly, the case study and the community set-up are artificial. Next, deterministic supply and demand functions and willingness-to-pay are used to conduct the study. Lastly, grid topologies are neglected and power losses are not taken into account directly. However, the modeling approach allows network topologies to be considered using the distance factor d _i,j_.”

# Reviewer report: Reviewer response for version 1

**DOI:** 10.21956/openreseurope.15465.r28328

**Published:** 2022-01-25

**Authors:** Sonja Wogrin, Lia Gruber

**Affiliations:** 1 Institute of Electricity Economics and Energy Innovation (IEE), Graz Universitz of Technology, Graz, Austria

Energy Communities are an important part of the energy transition, but still, a lot of questions on this topic need to be answered before its widespread application is possible. Peer-to-peer trading and dynamic participation are two important aspects of energy communities, which are addressed in this article. The article is well-written and easy to follow. The methodology proposed is novel and constitutes an original contribution to science.

 We have a couple of minor comments/suggestions for the authors to consider in in order to improve the publication:

- Ad 1.3 Method applied 2nd paragraph: “assuming that in the future (i) many people will already have PV modules and (ii) PV systems will be “mainstream products” and therefore installing a PV system is a low barrier for those interested in joining a local energy market. In particular, a community’s new member selection and decision-making process is the subject of interest.” That is a fair assumption to make but it also indicates that pure consumers can’t join as a new member while there are already ones in the old community. Later in the paper on the other hand it is possible since the possible PV peak output of the new prosumer is between 0 and 5 kW. This possibility could be clearly stated since energy communities are an important tool to fight the increasing problem of energy poverty, see JRC Report “Energy communities: an overview of energy and social innovation”(Caramizaru, 2019).
- Ad 2.3 Contribution beyond state-of-the-art: “optimize energy communities with peer-to-peer trading over the years” This is also described in the methodology but the case study is just one year. This makes sense since it is stated in 3.1.2 that it is just one year because of the focus on the selection process, but contradicts the contribution statement. We suggest clarifying this in the state of the art. 
  A related comment touches upon the title: even though the proposed methodology could be used in a dynamic way, the results only analyze static “1-year” cases. We suggest either changing the title to something like “deciding participation in local …” (and leaving out the dynamic part), or including a section where the participation process is evolving dynamically over a couple of years.
- Ad 3.1.1 Modeling framework: “Participants in the community are either households or small-to-medium-sized enterprises.” Here it would be interesting to add why? If the reason for this assumption is the Austrian legislature and/or that the load profiles are different of households and businesses, then this should be added.
- Ad 3.2: Even though it has been stated previously, it would improve the paper to point out again what is the upper level (objective and variables) and what is the lower level. Currently, section 3.2 starts with the definition of several objective functions and it was not until the definition of (8) that the order was clear to me.
- Objective function (8a) and (5). This constitutes a weighted average over costs and emissions. In the “pure” cases, where all members consider costs (or emissions), this function is sound. However, in the hybrid cases (with alpha between 0&1), costs and emissions are hard to compare, especially because they have very different units that might also have different orders of magnitude. It would improve the paper to either discuss how these functions have been normalized or to include a justification of how the hybrid cases might make sense.
- Ad 3.2.2 Community welfare: Part I community’s self-consumption – Maximizing CW would mean maximizing part I, therefore, maximizing sales to the grid (qtGout) and minimizing buying from the grid (qtGin). This leads to minimizing the cost and not maximizing the self-consumption. For the maximization of the community’s self-consumption, an incentive in the objective function is needed to consume as much of the produced energy in the community itself. Here the opposite is the case since the sales to the grid are maximized. Therefore, calling part I “self-consumption” is a bit misleading.
- Ad 3.3.2 Input data: Why do you use average retail electricity and spot market prices? This data is publicly available in hourly resolution. Model results will be more interesting when prices vary over the day.
- Ad Figure 4. It would improve the readability of the paper if the distance factors (how they are calculated in real-life) were to be explained briefly.
- Ad 4.4 Fairness measures: It would improve the paper if the authors could explain why this is a good definition of fairness in this case.
- Ad 4&5 Results & Sensitivity analyses: The result section is on the descriptive side. More conclusions and interpretations from a regulatory point of view would be interesting. You have a lot of results, which are described very thoroughly but what do you conclude from them? Especially in connection with your input. 
- With respect to the willingness to pay. How could one attempt to estimate such a parameter in real life?

**General comments:**

- The energy community members decide which new members to accept. Is it possible that some kind of market power issues (within the community) can arise in a way that large members can “over-rule” smaller members?
- It would be interesting to compare the results from model (8) to another model that is a single-level optimization model, that is essentially (8) except (8e). Meaning that you don’t have to take KKT conditions of the lower level. You just consider lower level constraints. So the community would be choosing new members maximizing (8a) without having a lower level objective function. How would results differ from what you currently have?

**Typos:**

- Ad 4.4 Fairness measures: “Fairness measures are no introduced” now instead of no.
- Ad Nomenclature: Parameter descriptions ptGin and ptGout are switched

Is the study design appropriate and does the work have academic merit?

Yes

Is the work clearly and accurately presented and does it cite the current literature?

Yes

If applicable, is the statistical analysis and its interpretation appropriate?

Yes

Are all the source data underlying the results available to ensure full reproducibility?

Yes

Are the conclusions drawn adequately supported by the results?

Yes

Are sufficient details of methods and analysis provided to allow replication by others?

Yes

Reviewer Expertise:

Modeling and optimization in the energy sector; bilevel programming; energy communities

We confirm that we have read this submission and believe that we have an appropriate level of expertise to confirm that it is of an acceptable scientific standard.

# Response

**Published:** 2024-10-15

**Authors:** Theresia Perger

**Affiliations:** TU Wien, Austria

**We want to thank the reviewer for their time and effort. We appreciate the comments and incorporated them in our updated version. Please see below the comments and our answers. **   Energy Communities are an important part of the energy transition, but still, a lot of questions on this topic need to be answered before its widespread application is possible. Peer-to-peer trading and dynamic participation are two important aspects of energy communities, which are addressed in this article. The article is well-written and easy to follow. The methodology proposed is novel and constitutes an original contribution to science. We have a couple of minor comments/suggestions for the authors to consider in in order to improve the publication:

- Ad 1.3 Method applied 2nd paragraph: “assuming that in the future (i) many people will already have PV modules and (ii) PV systems will be “mainstream products” and therefore installing a PV system is a low barrier for those interested in joining a local energy market. In particular, a community’s new member selection and decision-making process is the subject of interest.” That is a fair assumption to make but it also indicates that pure consumers can’t join as a new member while there are already ones in the old community. Later in the paper on the other hand it is possible since the possible PV peak output of the new prosumer is between 0 and 5 kW. This possibility could be clearly stated since energy communities are an important tool to fight the increasing problem of energy poverty, see JRC Report “Energy communities: an overview of energy and social innovation”(Caramizaru, 2019).

Thank you for this comment. It is true that pure consumers are not likely to be selected to an energy community if there are already many pure consumers in the old community. The energy community needs sufficient own generation to share within the community. Assuming that many households will own PV systems, there is more possibility that they are willing to share with consumer only households.We think that some communities will even prefer that pure consumers join, as shown in the results.

- Ad 2.3 Contribution beyond state-of-the-art: “optimize energy communities with peer-to-peer trading over the years” This is also described in the methodology but the case study is just one year. This makes sense since it is stated in 3.1.2 that it is just one year because of the focus on the selection process, but contradicts the contribution statement. We suggest clarifying this in the state of the art. 

This is correct. The text is reformulated to clarify the contribution: “ A method is developed based on the peer-to-peer allocation mechanism presented in 6 to optimize energy communities with peer-to-peer trading, including the annual entry of new members.”

 A related comment touches upon the title: even though the proposed methodology could be used in a dynamic way, the results only analyze static “1-year” cases. We suggest either changing the title to something like “deciding participation in local …” (and leaving out the dynamic part), or including a section where the participation process is evolving dynamically over a couple of years. Thank you for this suggestion. Nevertheless, we decided to keep the title as it is, because we want to point out the difference to other analyses on energy communities, which use a static approach instead. Our follow-up research included a process involving over the years, based on the model presented here.  

- Ad 3.1.1 Modeling framework: “Participants in the community are either households or small-to-medium-sized enterprises.” Here it would be interesting to add why? If the reason for this assumption is the Austrian legislature and/or that the load profiles are different of households and businesses, then this should be added.

We added two sentences to better explain our choice. “Different consumer types are considered to show a wider variety of load profile types in the analysis. Along with households, small businesses are also a key target group for RECs.”  

- Ad 3.2: Even though it has been stated previously, it would improve the paper to point out again what is the upper level (objective and variables) and what is the lower level. Currently, section 3.2 starts with the definition of several objective functions and it was not until the definition of (8) that the order was clear to me.

As suggested, we reformulate the text in Section 3.2.2 “In this work, the aim of peer-to-peer electricity trade is to maximize community welfare, which is the objective function of the lower-level problem.“ and in Section 3.2.3 “The cost-emission function CE, which is the objective function of the upper-level problem, is defined next.” 

- Objective function (8a) and (5). This constitutes a weighted average over costs and emissions. In the “pure” cases, where all members consider costs (or emissions), this function is sound. However, in the hybrid cases (with alpha between 0&1), costs and emissions are hard to compare, especially because they have very different units that might also have different orders of magnitude. It would improve the paper to either discuss how these functions have been normalized or to include a justification of how the hybrid cases might make sense.

You are right, there are different units in the objective function. In practice, costs (in EUR) and emissions (in tCO2) are often compared, e.g. when it comes to carbon prices, CO2 taxes, etc. 

- Ad 3.2.2 Community welfare: Part I community’s self-consumption – Maximizing CW would mean maximizing part I, therefore, maximizing sales to the grid (qtGout) and minimizing buying from the grid (qtGin). This leads to minimizing the cost and not maximizing the self-consumption. For the maximization of the community’s self-consumption, an incentive in the objective function is needed to consume as much of the produced energy in the community itself. Here the opposite is the case since the sales to the grid are maximized. Therefore, calling part I “self-consumption” is a bit misleading.

As the formulation is indeed misleading, we reformulate the description of part I of the community welfare to “Part I of community welfare measures the optimal resource allocation at the community level. In other words the community minimizes the electricity bill from the retailer. “

- Ad 3.3.2 Input data: Why do you use average retail electricity and spot market prices? This data is publicly available in hourly resolution. Model results will be more interesting when prices vary over the day.

Thank you for this suggestion. We chose to use average prices instead of hourly rates, as the vast majority of consumers still rely on fixed tariffs.

- Ad Figure 4. It would improve the readability of the paper if the distance factors (how they are calculated in real-life) were to be explained briefly.

We added a footnote to Section 3.2.1 to explain more details about the distance factor. “Network topologies must be taken into account when calculating electrical distances. The distance factor is specified in this paper on a scale from zero to one. Zero corresponds to the shortest distance, i.e. the distance from the prosumer to itself. One corresponds to the longest electrical distance in this part of the grid. Power losses are not taken into account directly. The grid losses are charged to the consumers via the grid tariffs in their electricity bill.” 

- Ad 4.4 Fairness measures: It would improve the paper if the authors could explain why this is a good definition of fairness in this case.

Thank you for this comment. We added explanations to the fairness measures. “The three fairness measures represent different characteristics of the energy community. QoS measures how fairly electricity trading is distributed, QoE compares the relative electricity costs of members, and MinMax assesses whether there is an imbalance in the amount of electricity members purchase from external sources, such as their individual retailers, outside the community. Those measures are interesting for members of energy communities so that they can assess how equally or unequally some aspects such as costs or trading volumes are distributed in the community.”

- Ad 4&5 Results & Sensitivity analyses: The result section is on the descriptive side. More conclusions and interpretations from a regulatory point of view would be interesting. You have a lot of results, which are described very thoroughly but what do you conclude from them? Especially in connection with your input. 

The conclusions and interpretations can be found in Chapter 6.  

- With respect to the willingness to pay. How could one attempt to estimate such a parameter in real life?

Community members should be well informed and can decide on their individual willingness-to-pay. General comments:

- The energy community members decide which new members to accept. Is it possible that some kind of market power issues (within the community) can arise in a way that large members can “over-rule” smaller members?

Thank you for this question. With the objective function proposed in Eq. (8a), members with a large delta in costs or emissions have more influence on the decision than others. It does not mean that there is an issue with market power of some members. It rather means that if a member is strongly affected by the addition of a new member to the community, it counts more than members who do not feel any changes. 

- It would be interesting to compare the results from model (8) to another model that is a single-level optimization model, that is essentially (8) except (8e). Meaning that you don’t have to take KKT conditions of the lower level. You just consider lower level constraints. So the community would be choosing new members maximizing (8a) without having a lower level objective function. How would results differ from what you currently have?

We did not run the analysis without Eq. (8e). We assume that it is not possible to leave out the objective function of the lower level problem, since electricity prices and willingness-to-pay are necessary to allocate electricity within the community. Typos:

- Ad 4.4 Fairness measures: “Fairness measures are no introduced” now instead of no.

The typo is corrected.

- Ad Nomenclature: Parameter descriptions ptGin and ptGout are switched

The typo is corrected.
